# Changing activity behaviours in vocational school students: the stepwise development and optimised content of the ‘let’s move it’ intervention

**DOI:** 10.1080/21642850.2020.1813036

**Published:** 2020-09-27

**Authors:** Nelli Hankonen, Pilvikki Absetz, Vera Araújo-Soares

**Affiliations:** a Faculty of Social Sciences, University of Helsinki, Helsinki, Finland; b Department of Public Health and Clinical Nutrition, University of Eastern Finland, Joensuu, Finland; c Institute of Health and Society, University of Newcastle, Newcastle, UK

**Keywords:** Intervention development, complex intervention, theory-based intervention, Self-Determination Theory, reasoned action approach

## Abstract

**Background:** School-based interventions that increase physical activity (PA) in a sustainable way are lacking. Systematic and participatory, theory and evidence-based intervention development may enhance the effectiveness of complex behavioural interventions in the long term. However, detailed descriptions of the intervention development process are rarely openly published, hindering transparency and progress in the field.

**Aims:** To illustrate a stepwise process to develop intervention targeting PA and sedentary behaviour (SB) among older adolescents, and to describe the final, optimised version of the intervention, detailing content of sessions by theoretical determinants and techniques.

**Methods:** Two established intervention development frameworks (Intervention Mapping and Behaviour Change Wheel) were integrated, leading to a comprehensive evidence and theory-based process. It was informed by empirical studies, literature reviews, expert and stakeholder consultation, including scenario evaluation and component pre-testing. In all steps, contextual fit and potential for sustainability were ensured by stakeholder engagement.

**Results:** As a large majority of youth opposed decreasing screen time, increasing PA and decreasing SB were defined as target behaviours, with peers and the school context including classroom practices as key social environments in influencing youth PA (problem specification, step 1). Behavioural diagnosis (step 2) identified a variety of determinants in the domains of capability (e.g. self-regulation skills), motivation (e.g. outcome expectations) and environmental opportunities. These were organised into an intervention theory integrating several formal theories, including Self-Determination Theory. Theory-aligned principles guided material design (Step 3). Feasibility RCT allowed optimisation into a final intervention protocol (step 4).

**Conclusions:** Intervention elements target students directly, and indirectly by changing teacher behaviour and the school and wider environment. A systematic development and optimisation led to a high potential for sustainability. The detailed intervention content, with specification of the hypothesised mechanisms, allows for other researchers to replicate, adapt or refine parts or the whole intervention, considering specific target groups and (sub-)cultures.

## Background

Despite the benefits of physical activity (PA), adolescents worldwide engage in far less PA than is recommended (Hallal et al., 2012), with the trend being stronger among those with lower socioeconomic status (SES) (Van Der Horst, Paw, Twisk, & Van Mechelen, 2007). The Finnish National Recommendation for youth physical activity includes light PA (LPA) and moderate to vigorous PA (MVPA), and as one key form of sedentary behaviour (SB), screen time limited to maximum of 2 h daily. Reflecting the SES difference, the prevalence of meeting PA recommendations is about 50% lower and prevalence of overweight almost 50% higher in Finnish vocational schools than in high schools (National Institute of Health and Welfare (THL), 2011), and it tends to decline during adolescence (Van Der Horst et al., 2007).

Schools are optimal settings for youth PA promotion (van Sluijs, McMinn, & Griffin, 2007): they reach a majority of potential participants, who also spend most of their time in school context. However, school-based interventions have mainly shown short-term effects on MVPA (Dobbins, DeCorby, Robeson, Husson, & Tirilis, 2009), variance in effect sizes has been high (Crutzen, 2010) and gender differences in effectiveness have favoured girls (Yildirim et al., 2011). Generally, more intensive and longer interventions have been more effective (Oldenburg, Absetz, & Chan, 2010). Furthermore, school-based PA interventions have mainly focused on children, and interventions among older adolescents or in vocational schools are rare (van Sluijs et al., 2007).

When the present study was started, only 10 RCTs of school-based interventions to improve PA or SBs among 15–19-year-olds had been reported (Hynynen et al., 2016). Furthermore, few of the studies had measured behaviour change over a longer follow-up, and the few that had, showed no effects. None explained in detail the intervention development process, even though half of them mentioned a behavioural theory as a basis of the intervention, half briefly described some stages of what could be interpreted as a development process, but only one study named a specific intervention development framework that they used. Thus, there is a demand to carefully develop and report studies in this area.

PA interventions should be simple, effective, generalisable, realistic and situation specific. School-based programmes should be suitable for the school environment and designed to be implemented by teachers (Lubans & Sylva, 2009; Reilly & McDowell, 2003) and hence include both teacher- and student-level intervention strategies. For sustainability and scalability, they should be low cost, embeddable into the existing school structures, with good fit with the context and the daily practices and the values of the target group.

The UK Medical Research Council Guidance for complex interventions (Campbell et al., 2000; Craig et al., 2008) recommends using a phased approach in designing complex multi-level interventions, but utilisation of intervention development frameworks in practice is rarely described in the literature. We aimed to develop a whole-school system intervention to promote PA and to reduce SB among Finnish vocational school students with interventions at teacher and student level. This paper describes in detail the development of the student-level intervention, guided by insights from the UK MRC Guidance and established frameworks for intervention design. Development of the teacher intervention is only briefly referred to as a more detailed description of it can be found elsewhere (Köykkä, Araújo-Soares, Sniehotta, Knittle, & Hankonen, 2019).

We report the stepwise development process, including four steps from problem analysis (Step 1) to definition of the scientific core (Step 2), the design of the intervention materials (Step 3) and empirical optimisation of the programme (Step 4) (Araújo-Soares, Hankonen, Presseau, Rodrigues, & Sniehotta, 2018). The full final intervention content is also presented.

## Methods

### Context

Educational level is the most usual marker of socioeconomic position (SEP) in Finland (Lahelma, Martikainen, Laaksonen, & Aittomäki, 2004). A publicly funded compulsory comprehensive school is provided to all children until age 15 (Grade 9), after which they apply to an average three-year secondary education either in high schools (academic track) or vocational schools (occupational training). High schools and vocational schools are typically situated in completely separate school buildings and are organisationally independent from each other. This educational divide is a major predictor of socioeconomic health inequalities in adulthood (Lahelma et al., 2004), which are among the highest in the OECD countries (Kunst et al., 2005). Lower educated populations have sometimes been found to respond to health promotion less favourably than the higher educated (e.g. Verloigne et al., 2011).

For a whole-school intervention targeting those with lower SEP, vocational schools are the first possible point. They are organisationally independent from high schools, and typically in different locations. Curriculum consists of both hands-on teaching in workshop-type classrooms and traditional, more sedentary classroom instruction (e.g. language classes). A small amount of health education and physical education (PE) is compulsory (usually one 40-hour course over 2–4 months).

### Procedures

The intervention development process was in line with the UK Medical Research Council Guidance recommendations (Campbell et al., 2000; Craig et al., 2008), using a phased approach to design and feasibility/pilot testing. For more specific guidance, we used the Behaviour Change Wheel (BCW) and the Intervention Mapping (IM) approach as integrated into four steps (for an analysis and integrative overview of frameworks, see Araújo-Soares et al., 2018), applying multiple theories and mixed methods. Four steps were followed in the development: In step 1 the problem was analysed and intervention objectives were developed; step 2 allowed for the definition of the behavioural scientific core of the intervention; step 3 led to the design/development of the intervention materials and finally; step 4 consisted of an empirical optimisation using a randomised feasibility study. Table 1 shows these frameworks mapped on to each other, according to the key tasks of intervention development (Hankonen & Hardeman, 2020).

**Table 1.** Stages and steps of IM and BCW frameworks mapped onto key intervention development tasks (adapted from Hankonen & Hardeman, 2020).

	Intervention development tasks	Integrative review (Araújo-Soares et al., 2018)	Intervention mapping (Bartholomew Eldridge et al., 2016)	Behavior change wheel (Michie et al., 2014)
**Problem**	**Task 1.** What is the problem to be addressed?	A. *Analysing the problem and developing an intervention objective*	Step 1: *Logic model of the problem*	Stage 1: *Understanding the behaviour*
**Intervention**	**Task 2.** What are the hypothesised mechanisms of effect on behavior and intervention components?	B. *Defining the scientific core of the intervention* Causal modelling Defining intervention features • Developing a logic model of change	Step 2: *Programme outcomes and objectives – Logic model of change* Step 3: *Programme Design*	Stage 2: *Identifying intervention options* Stage 3: *Identifying content and implementation options*
**Materials**	**Task 3.** Development of intervention materials and technology	C. *Development of material and interface*	Step 4: *Programme production* Step 5: *Implementation plan*	
**Testing and optimisation**	**Task 4.** Empirical optimisation of the intervention	D. *Empirical optimisation*	See Step 4	
**Evaluation**	**Evaluation task:** Is the intervention effective? What are the processes involved?	E. *Evaluating the intervention* F. *Process evaluation*	Step 6: *Evaluation Plan*	
**Implementation**	**Implementation task:** How to implement it in the ‘real world’?	G. *Implementation: real-world application*	(Step 5: Implementation plan)	

With regard to behavioural theories, the COM-B model was used as a general model of behaviour, including the Theoretical Domains Framework (TDF) (Cane, O’Connor, & Michie, 2012; Michie et al., 2005), that allows a comprehensive diagnosis of behavioural influences without commitment to particular theories. However, more specific theories (e.g. Self-Determination Theory (SDT), Reasoned Action Approach (RAA); Habit Theory (HT), Control Theory (CT)) were drawn on to inform specific aspects of the intervention, as well as empirical studies conducted in preparation for intervention development. In making decisions (e.g. links to intervention objectives, determinants, behaviour change techniques i.e. BCTs), the key criteria were Acceptability, Practicability, Effectiveness, Affordability, Side-effects, Equity (Michie, Atkins, & West, 2014) and Changeability (Bartholomew, Parcel, Kok, Gottlieb, & Fernández, 2011) (APEASE-C). More information on the logic/theoretical model will be presented below (Results, Steps 1 and 2).

In addition to the core intervention development team, the process involved a stakeholder and steering group, including key stakeholders and experts: researchers, teachers, PA and SB experts. A student panel collaborated alongside the core team and was consulted at key stages. Work with the target group and stakeholders included following elements:

- Consulting experienced PE and other teachers to obtain the existing feasible practices and ideas for PE teaching, and for SB reduction in classrooms, as well as creation of innovative intervention activities
- Student panel: Hands-on pre-testing of newly created intervention activities for lessons
- Activities in vocational school classrooms: Hands-on pre-testing of sitting reduction strategies
- Intervention ideas and scenarios brainstormed with and later evaluated and ranked by expert group (stakeholders, steering group, experts)
- Engagement with health promotion organisations’ experiences and know-how in the health promotion opportunities and contextual elements relevant to intervening in vocational schools

*Step 1 Methods: Analysing the problem*

In order to analyse the problem comprehensively, we undertook several sub-studies and activities. The COM-B model was used as a general theoretical framework, and more specific theories (see above) were used for the assessment and identification of behavioural determinants. A belief elicitation study (unpublished) that formed a basis for a survey on determinants of PA and SB among students (SOLE/Alias Survey: *N *= 765) was conducted (Hankonen, Heino, Hynynen, et al., 2017; Nurmi, Hagger, Haukkala, Araújo-Soares, & Hankonen, 2016). A seven-day objective PA measurement was carried out within a sub-sample (*n *= 69) in order to validate survey self-report measures and confirm the feasibility of accelerometry in the target group. Low-active students were defined as those who self-reported engaging in more than 20 min of PA on less than three days a week. A writing contest for older adolescents aimed to acquire narratives about their own perceptions of critical incidents leading to PA changes over the course of their life, as well as material for the intervention. Also, we conducted qualitative interviews, to better understand how adolescents perceive the role of activity behaviours in their daily lives and key influences, and reviews and systematic reviews of literature on interventions and approaches to effectively intervene on youth PA and SB. Data from all these sources were triangulated and led to evidence-based constraints that shaped intervention development choices. In the beginning, informal interviews with vocational school directors and teachers helped us gain insight into the needs and resources of schools and staff, as well as insight into the specifics of the context.

*Step 2 Methods: Defining the behavioural scientific core of the intervention*

The BCW (Michie et al., 2014) and IM (Bartholomew et al., 2011) were used to shape the work by the core intervention development team. A wider stakeholder team of experts and other stakeholders was formed using IM guidance (2011), and included experts in the domain of PA and SB (UKK Institute, LIKES Research Centre, National institute for Health and Welfare THL), PE teachers (both from collaborating vocational schools and teacher association), teacher education experts (Jyväskylä University, Continuing Professional Development Unit for PE teachers), health promoting NGOs (working within vocational schools), students (national student organisation of vocational school students) and the research team.

Based on evidence gathered in Step 1, we assessed the needs and strengths of the target group and school setting; defined intervention objectives; and selected relevant determinants (mediators of intervention effect) and BCTs to change the determinants. Alternative intervention scenarios and individual smaller elements (e.g. standing desks in classrooms, possibility to lend PA equipment at school, reviewing PA goals in small groups, contacts between schools and local PA actors) were subject to evaluation and further development.

*Step 3 Methods: Designing and developing intervention materials*

Based on the theoretical model of the intervention and the specific BCTs identified in step 2, an advertising agency was commissioned to design all intervention materials. Refinements to the proposed designs were made based on behavioural science evidence (e.g. eliminate messages reinforcing appearance-based motives to be physically active, in light of the possible adverse effects on well-being and high-quality motivation). In this process, and to assure higher levels of acceptability and feasibility of materials, student engagement was intensified.

*Step 4 Methods: Empirical optimisation*

A randomised feasibility study was conducted (ISRCTN34534846) to pre-test intervention activities and research procedures in one vocational school unit, with 16 teachers and 42 student participants (Hankonen, Heino, Hynynen, et al., 2017). Optimisation of *research procedures* has been reported in a separate paper (Hankonen, Heino, Hynynen, et al., 2017). Optimisation of the *intervention procedures* was based on field notes by the researcher implementing the intervention, feedback received from study participants on sessions and materials as well as interviews with low-active students (*n *= 15). For the most pressing issues and potential pitfalls for intervention optimisation, the expert group (see Step 3) brainstormed potential solutions and necessary requirements. The core intervention development team then utilised this feedback to finalise intervention content and form.

The above-described development work was conducted in practice over several months, and included many activities.

### Ethics statement

Participants of empirical studies were treated according to principles of the Helsinki Declaration, and were informed about their right to withdraw from the study at any point. An Ethics Committee of the Hospital District of Helsinki and Uusimaa reviewed the study protocols and provided favourable views.

## Results

**Figure 1.**
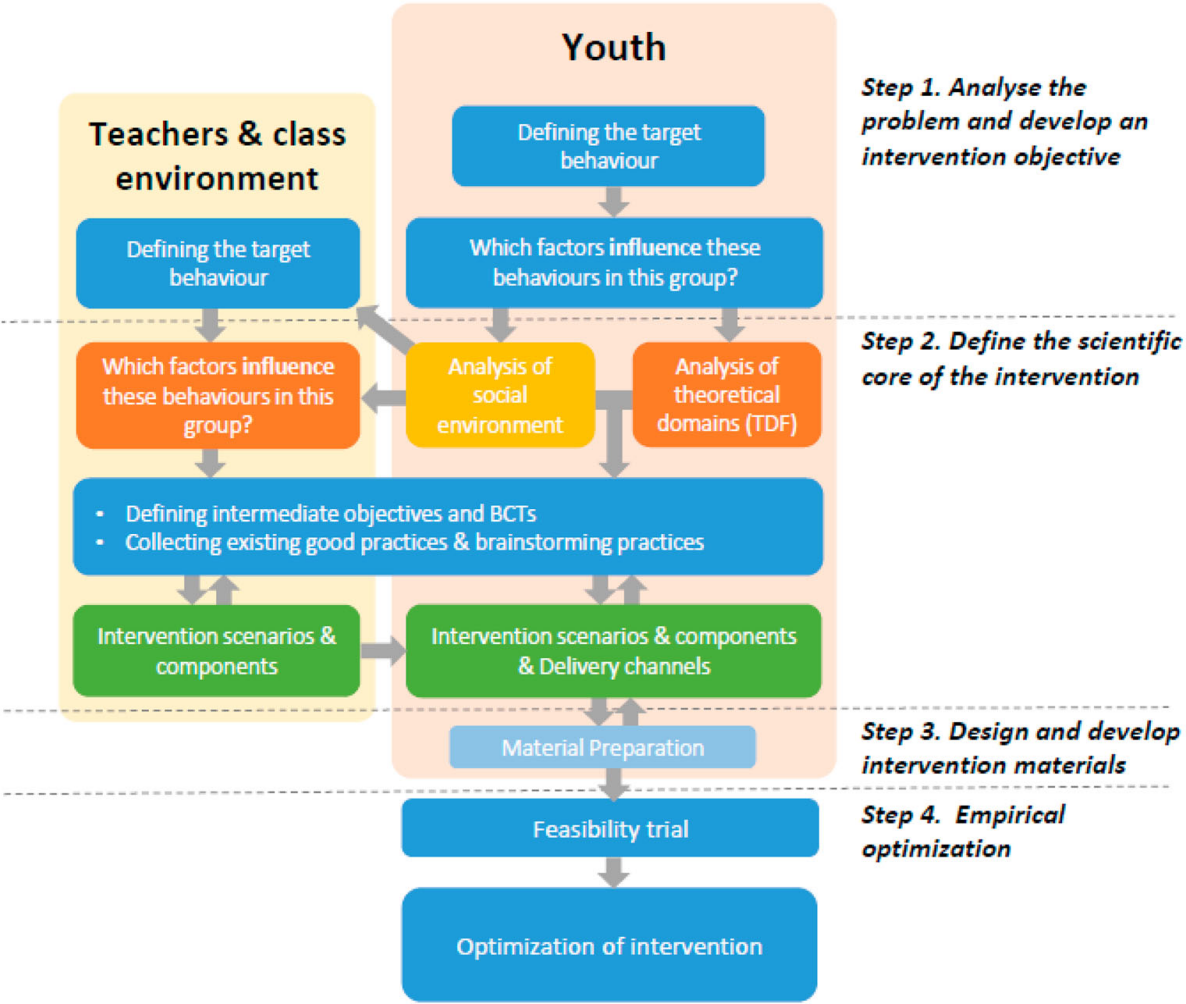
Intervention development process overview.

Intervention development was an iterative process as usual (Bartholomew, Parcel, Kok, Gottlieb, & Fernandez, 2011; Michie, van Stralen, & West, 2011) with empirical data obtained at different steps from different sources, feeding back and shaping intervention decisions, and often determining going back a step in order to reach a final draft intervention. Figure 1 shows the main steps of the development process (For an in-depth introduction to the integrated steps, see Araújo-Soares et al., 2018).

### Step 1: Analysing the problem

To analyse the problem and understand the target behaviour and context, findings from qualitative and quantitative studies and systematic review were synthesised to identify and specify the intervention objectives and determinants of behaviour and behaviour change. We also assessed needs and community capacity. Supplementary Table S1 collates all studies.

#### Selecting and specifying the target behaviour

*Qualitative interviews* shed light on how girls and boys in vocational schools engaged and perceived PA and existing opportunities for PA (Liimakka, Jallinoja, & Hankonen, 2013) and their perceptions of influences on changes in PA over the life course (Liimakka, Jallinoja, & Hankonen, in press). It also revealed that some students may equate PA with PE classes (in schools) and with MVPA, pointing to the need to address this misconception and clearly define these behaviours for (and with) students. There was wide variation in how students experienced PA. A belief elicitation study identified most salient beliefs (Table 2).

**Table 2.** Key findings from preliminary research phase. See supplementary Table S1 for the original studies.

Step 1: 1. Gender differences in motivation to increase PA were observed, with girls being more motivated than boys. 2. Willingness to increase PA was promising – especially among low-active girls. However, gender differences between the ‘acceptability’ of this intervention objectives point to a need to include in the intervention both motivational and post-intentional behaviour change strategies. 3. Key salient beliefs regarding outcomes of PA included advantages such as better physical condition, better mood and weight management, and also disadvantages such as tiredness, lack of energy, less time for other things. 4. Injury prevention was identified as one key component to be included in PA promotion interventions, and in this age group, the recommended way to incorporate injury prevention was to improve motor-coordination abilities [e.g. 28]. 5. PA takes varied forms for each individual, hence it will not be feasible to specify common MVPA objectives for all participants (e.g. commuting or leisure PA, types of PA). Based on theory, evidence and pre-testing, we identified a need for personal agency in planning SMART goals in a personalised way so that they would be attainable and relevant for participants. Hence, for PA individual goal setting needs to be facilitated. SB, however, needs to be addressed within the classroom setting given students’ ‘red lines’ i.e. low acceptability of leisure screen-time reduction. 6. Teachers perceived sitting reduction as a useful and acceptable way of organising the lesson, and often even necessary, because students were seen as unable to concentrate if required to sit still the whole time [28]. 7. Any intervention in the school setting needs to have high fit with the curriculum and not take up too many lessons (see Supplementary Material for more detailed information on fitting the programme in the national curriculum). Step 2: 8. Behavioural diagnosis rendered all COM-B domains relevant except for the physical capability. 9. Previous similar interventions are heavily focused on volitional strategies, i.e. goal setting and action planning [9], which might imply that techniques to increase motivation are too scarce. Autonomous PA motivation is related to the use of self-regulatory strategies, which, in turn, predict PA [22]. Hence, as evidence showed that levels of motivation of this age group students to increase their PA are quite low but that prior interventions had little focus on motivation, we concluded that in this context and target group, motivation may have been under-targeted relative to target group’s needs. 10. Previous interventions have shown an effect on girls but not on boys. Our original study showed that girls were at the outset more motivated to change their PA than boys. Lack of components targeting motivational aspects and focus on post-intentional self-regulation strategies in previous research may account for the finding that boys are less likely to change in many of the school-based interventions [e.g. 4].

*The SOLE/Alias survey* investigated PA and predictors of PA (see also Step 2). Results on PA levels were in line with the national school health survey. Regarding motivation to increase PA, altogether 42.7% of the students indicated that they are happy with their current level of PA with no willingness to increase it. Among low-active students, there was a significant gender difference with 75.4% of girls willing to do more PA compared to only 44.6% of the boys (CI95 of population difference: [19.7, 41.9] %-points).

The majority (58.7%) was satisfied with the levels of their screen time. Even among those with over 4 h of screen time per day during weekdays, 55.2% stated being happy with the current levels. However, some acknowledged that screen time would reduce their opportunities for PA and spending time outdoors. Only few (12.2%) gave a correct answer regarding the national PA recommendation. Over one-third (35.8%) reported the screen time recommendation correctly at 2 h, and a similar proportion underestimated it (1 h/day). Awareness of the recommendations was not associated with motivation to change behaviour (Hankonen, Heino, Kujala, et al., 2017).

As most of the target group reported no willingness to change their exposure to screen time, the changeability of this target behaviour was estimated to be low. Hence, instead of the Finnish National Recommendation of reducing screen time, we selected two other behavioural objectives for SB: reducing SB in school and elsewhere.

#### Specifying determinants for change

The qualitative interviews (Liimakka et al., in press, 2013) pointed at social networks as a key influence on PA and sports (see Table 2). Behavioural determinants of PA and SB identified from the SOLE/Alias survey informed the selection of mediators of behaviour change (see Table 3 and supplements: Figure S2, Figure S3, Table S6, Table S7). To gain more in-depth understanding of the target group, we compared the vocational and high-school students. The results revealed small differences in 13 out of 17 determinants assessed (Hankonen, Heino, Hynynen, et al., 2017).

**Table 3.** Intervention’s programme theory, described as key theoretical determinants linked to intervention content.

Theoretical determinant	Corresponding TDF domain	Intervention content linked to determinant (incl. sample BCTs), examples:
Self-efficacy	Beliefs about capability	Verbal persuasion of capability (BCT 15.1) Skill provision, Instruction on how to perform behaviour (BCT 4.1) Graded tasks (BCT 8.7) (i.e. emphasis on moderate goal setting, (incl. principle ‘Adding any activity is good/Every little bit counts’)) Modelling (focus on encouraging stories), Demonstration of the behaviour (BCT 4.1) *Student sessions, workbook, posters, table stands*
Outcome expectations (both reflective and experiential); Knowledge	Beliefs about consequences Knowledge	Information about health consequences (BCT 5.1); social and environmental consequences (BCT 5.3), and emotional consequences (BCT 5.6), incl. principles ‘Any activity is better than nothing/Every little bit counts’ and ‘Sitting sucks’. Focus on communicating positively framed benefits of increased activity Behavioural experiments (sessions and homework) (BCT 4.4) *Student sessions, workbook, posters, table stands*
Autonomous motivation (intrinsic, integrated, identified motivation)	Goals	Autonomy supportive style across sessions and materials Principle ‘It is your choice’ (across sessions and materials) Emphasis on selecting personally important reasons (principle ‘Know what moves you’) Emphasis on selecting autonomous goals (incl. principle ‘Not fatless body but well-being’) Behavioural experiments (BCT 4.4) Identification of oneself as active (self-concept); Identity associated with changed behaviour (BCT 13.5) *Student sessions, workbook, posters, table stands*
Descriptive norms	Social influences	Information about others’ behaviour and attitudes towards PA (incl. information about others’ approval, BCT 6.3) *Student sessions, workbook, posters, table stands*
Intention	Intentions	All activities directed at the other determinants were hypothesised to increase intention
Self-regulation	Behavioural regulation	Goal setting (behaviour) (BCT 1.1), goal review (BCT 1.5), Discrepancy between current behaviour and goal (BCT 1.6) Action planning (BCT 1.4), coping planning (problem solving) (BCT 1.2) Self-monitoring of behaviour (BCT 2.3) *Student sessions, workbook, posters, table stands*
Environmental opportunities in school class	Environmental context and resources	Environmental changes in classroom (physical equipment) (BCT 12.1, 12.5) *PA equipment, e.g. gym balls, standing desks, gym sticks, Pilates cushions*
Environmental opportunities in school class	Environmental context and resources	Teacher activity in classroom (e.g. activity breaks) (BCT 12.1; 12.2) *Teacher workshops, teacher guide, website*
Environmental opportunities in school	Environmental context and resources	Better access to school PA facilities (BCT 12.1) *Improved access or improved awareness (informed via leaflets in student sessions)*
Environmental opportunities at home and neighbourhood	Environmental context and resources	Home workout videos (BCT 12.1; 12.5) Better access to neighbourhood PA opportunities (BCT 12.1) Social support *Provision of home workout videosImproved awareness of the existing opportunities at home, online, environmentArrangement of low-cost PA deals with community PA providers (informed via leaflets in student sessions)*

#### Ensuring fit of the selected strategies with the implementation context and potential for sustainability

In the early phases of the project, students in vocational schools were required to undertake 27 h of PE and 27 h of Health education (HE) during their three-year curriculum. These were previously organised as two separate courses, but due to curriculum changes by the later government this course provision was cut by over 50% of study hours (over three years only 27 h of both PE and HE). These topics are taught by qualified PE teachers, although currently a large part of the teachers are not formally qualified.

*A staff survey* (*N *= 301) informed us about PE classes, the availability of extracurricular activities and accessibility of PA facilities. We collected teachers’ views of candidate intervention strategies for sitting reduction (in classrooms i.e. specific behavioural objectives for teachers) with this survey and *focus group interviews* (Laine, Araújo-Soares, Haukkala, & Hankonen, 2017). (For the use of these findings in the development of the teacher intervention, see (Köykkä et al., 2019)).

### Step 2: Defining behavioural scientific core

#### Understand causal and contextual factors

We aimed to understand and identify contextual influences on PA and SB. Using the first version of the BCW guide (Michie et al., 2014), the core intervention development team first discussed and investigated the drivers of influences on behaviour. (See Supplementary Figure S1 for the social network map of influences on students PA and SB.) The behavioural diagnosis rendered all COM-B domains potentially important targets. The only subdomain that already had sufficient levels was deemed to be *physiological capability*, as all youth can be expected to have sufficient capabilities for non-professional, leisure-time PA. For more specific guidance on the processes to shift in order to change *motivation*, we selected SDT (Ryan & Deci, 2000) and RAA (Fishbein & Ajzen, 2011), and on *capability*, goal setting and control theories. Reasons for these choices related to evidence base in this population and behaviours (McEachan, Conner, Taylor, & Lawton, 2011; Michie, Abraham, Whittington, McAteer, & Gupta, 2009; Ng et al., 2012). For simplicity, constructs from these theories are presented under the TDF (Cane et al., 2012; Michie et al., 2005).

#### Develop a logic/theoretical model

Table 3 presents an overview of the main theoretical determinants of PA behaviour, along with examples of intervention content targeting them and the corresponding TDF domains. The hypothesised mechanisms for student PA change have been listed in OSF (https://osf.io/h2uaq/).

Considering the identified determinants of behaviour, the theoretical model of the intervention was first and foremost based on the SDT (Deci & Ryan, 2000), with constructs from other approaches such as RAA (Fishbein & Ajzen, 2011) and self-regulation and planning approaches were also used, but SDT corollaries were used as a form of making decisions on the determinants to target. For example, the RAA posits injunctive norms as important predictors of behaviour and thus intervention targets. SDT principles conflict with this hypothesis by positing that external pressure would contribute to controlled forms of motivation which, as opposed to autonomous motivation, would not lead to sustainable changes in behaviour. Therefore RAA injunctive norms were not included into the theoretical model while RAA descriptive norms were included.

#### Define Intervention Features

After being presented insights and evidence (e.g. student interviews), the expert group generated ideas of practical intervention strategies and techniques, followed by more specific analyses of strengths, weaknesses and preconditions for feasibility. Based on these (as well as evidence from the reviews and survey), the core intervention team elaborated on these and selected some of the generated ideas as potential intervention components, and created alternative intervention scenarios. In the next expert group meeting, experts assessed these intervention scenarios with regard to their potential efficacy and feasibility in the school setting. Divided in small groups, they discussed and listed each of the intervention scenario’s strengths, weaknesses, opportunities and threats, and gave further refinement suggestions. APEASE-C criteria were also considered. These were then further developed by the core team.

The intervention functions and policy categories (BCW) were partially pre-defined as the project was to a school-based intervention: hence, guidelines, legislation, finances, etc. were out of question. However, to plan for wider impact and dissemination, the National Board of Education was contacted early on to initiate discussions about the future possibility to integrate the Let’s Move It programme into the Finnish school curriculum, and to ensure fit with the current PE and health education curriculum.

In the design process, evidence-based elements of effective school-based health promotion programmes identified in reviews (Dadaczynski & de Vries, 2013; Peters, Kok, Ten Dam, Buijs, & Paulussen, 2009) were also incorporated (if in line with the core scientific model of this intervention as well as with its logic model and if feasible). Although these reviews targeted different age groups, upon detailed analysis, the conclusions seemed to be generalisable to older adolescents (see also Supplementary Table S2). These elements included the use of theory; addressing social influences; addressing motivations and cognitive-behavioural skills; training of and on-going support to facilitators; using multiple components targeting behaviour (e.g. combining education with environmental change); programme fit with school routines; engagement of target group; and collaboration with community partners. As using classroom-based education alone (Dobbins et al., 2009) and targeting multiple health behaviours (Crutzen, 2010) have been shown to be ineffective in promoting PA, we chose a multi-level approach targeting PA and sedentary behaviours in the school setting and also supporting students in changing their behaviours in other life contexts.

Our *systematic review* of previous similar RCTs coded the interventions for the BCTs and other elements to identify what characterises effective interventions (Hynynen et al., 2016). In line with PA interventions among adults, BCTs such as self-monitoring and goal-techniques, including graded tasks, were supported.

We considered a variety of intervention features but had to leave many candidate strategies out. For example, for student intervention, we considered *mental rehearsal* (of instigating PA session and overcoming barriers, and of positive consequences); *individualised PA report feedback and guidance*; *elements targeting behaviour change maintenance*, e.g. habit formation for PA and SB. However, due to too short slots, expenses and scalability concerns, the intervention core team decided to leave these out.

In selecting intervention features, the changeability of various key determinants was considered, relative to the resources available and practicability (along with other APEASE-C criteria). For example, friends were evaluated to be important (‘effectiveness’), however difficult to directly target and change (‘practicability’, ‘changeability’) in a school-based intervention (as for many vocational school students, they have an already established friends group elsewhere and do not mainly hang out with classmates). Therefore, we decided to teach ways in which the youth may get their friends involved in PA, but not target friends directly as it would be costly.

Practicability issues relative to evaluation design also affected some choices. For example, whole-school strategies such as a kick-off event for entire school along with intervention-relevant roles and tasks for entire staff (including janitors and cleaners) were considered, but arranging considerable school-wide support – especially when not all students would be included in the longitudinal RCT design – was deemed too costly and impracticable to carry out properly given the cRCT design with multiple schools.

As mentioned above, components of the intervention were pretested among student panel and teachers/classrooms with students. A panel consisting of low-active students was consulted to pre-test and generate ideas for refinement of potential practical sub-components of the intervention in several face-to-face sessions.

### Step 3. Design and development of intervention materials and production

Programme planning included testing of all programme components and materials with user involvement. The key principles also in designing the materials relied on a participatory approach. Results from the belief elicitation study and the subsequent survey were used to develop the intervention (e.g. messages for the sessions, poster campaign).

Material design was conducted in collaboration with an *advertisement agency*. Selected marketing agencies were invited to make an offer on providing a visual look for the intervention as well as designing catchy poster slogans to support memorability of the central messages. A shortlist of agencies was then invited to pitch their team and ideas. They were assessed by a minimum of 3 team members for several dimensions, e.g. their ability to produce credible content for the target group, their understanding of social marketing principles and the value for money. The agency was required to meet with the youth (student panel) at least twice to ensure target group input.

The research team supervised closely the advertisement agency’s design work and made sure that out of alternative designs and concepts, the ones aligned with the SDT and other principles were selected for further development. For example, visual looks and concept promoting an excessive focus on the looks and masculine muscular body were rejected out of the interest to avoid unintended side-effects such as increasing dysfunctional body dissatisfaction and thereby possible eating disorders or unhealthy ways of increasing muscle mass.

For *tri-fold brochures* that were to be set on the tables in school, e.g. cafeteria tables, we designed content that would further target key determinants of student sessions. We used the writing contest stories as inspiration to model changing one’s PA and coping with barriers on the way. The aims, key messages and key BCTs used in these posters and brochures are presented in Supplementary Table S3 and S7.

To increase the appeal of the intervention to youth, we also reached out to celebrities (e.g. comedians and musicians) that would be able to pose as champions and support the messages, but despite several contacts, and some candidates considering participation, none of them volunteered in the end.

Home workout videos was one of the ways of increasing both opportunities and skills for PA, with an instructor showing ways to conduct PA activities in the home environment, without need for special equipment, suitable for people with varying levels of physical fitness. The production was manifold. First, a collaboration with a *university of applied sciences’ class* resulted in sample exercises produced by the students within a course, then *two young personal trainers* were asked to produce videos, but finally, the videos were designed by an in-house staff member (with master’s degree in social psychology and also a personal trainer certificate). The videos were designed to allow ‘self-tailoring’: the 5–10-minute videos could be combined to suit one’s wishes and needs, and also, combined with any music one prefers, as the video contained only some oral explanation in the background (taking into account positive atmosphere and low-active youth’s perspective).

The input of target group was relevant in all steps, also this one. For example, the alternative intervention names were subject to student vote, leading to the selection of the name, Let’s Move It.

### Step 4. Empirical optimisation

In the feasibility trial, we detected high willingness of students to participate in the trial and intervention, and acceptability of the concept (Hankonen, Heino, Hynynen, et al., 2017). We distinguished systematically issues requiring modification within the intervention as well as in the research procedures (Bugge et al., 2013). The issues identified and the modifications carried out are reported in Supplementary Table 8.

We also specified more closely intervention behavioural objectives: For SB, for the teacher intervention, these were breaking up sitting and reducing total amount of sedentary time in the classrooms. When assessing students about social cognitions relative to these behaviours, we formulated the questions to refer to restricting i.e. limiting one’s SB (and not reducing one’s SB). PE teachers’ feedback was taken into account to modify the intervention so that it could be delivered by health and PE teachers, although in the research, this was done by trained team members.

We also made adjustments based on what BCTs we envisaged the participants to ideally enact and when (enactment fidelity, Bellg et al., 2004). The table in Supplementary Figure S2 was created in 2014 to help get insight on the expectations for BCT enactment the intervention would place on the participants over the course of the intervention period. This helped looking at the delivered BCTs from the perspective of the participants, including the burden. For example, it was noted that enacting PA self-monitoring weekly would become quite heavy for some; thus, it was differentiated on which weeks self-monitoring (i.e. PA diary) was more important and when optional, to make the expectations more realistic and practicable.

#### Main conclusions from Step 4: The final intervention

**Figure 2.**
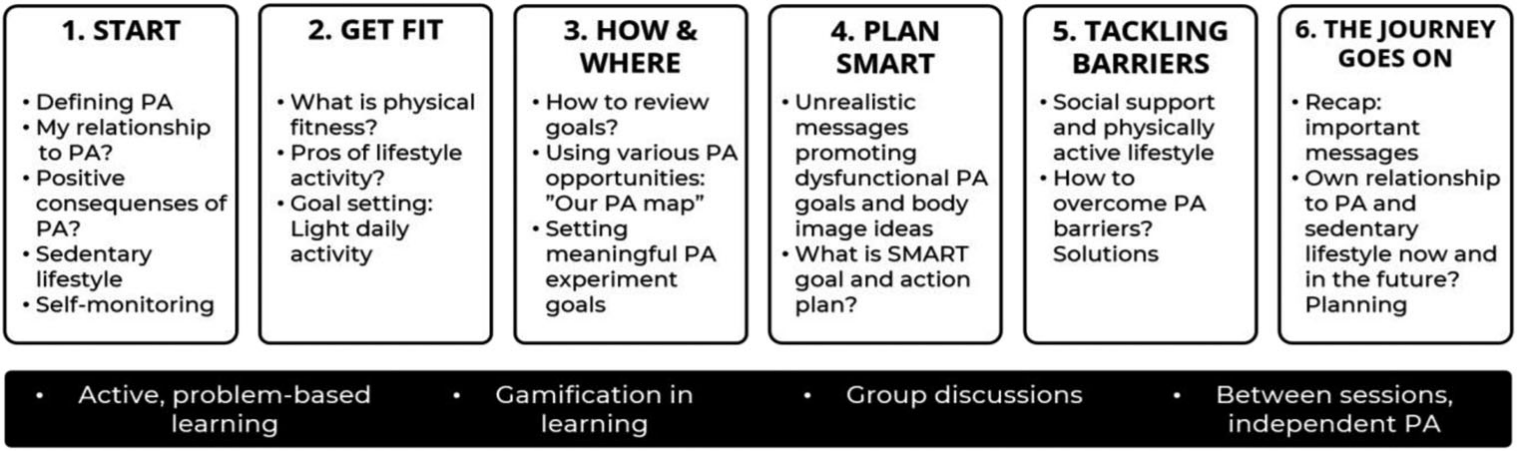
Overview of the Let’s Move It student intervention sessions.

This paper elaborates the brief intervention description of the RCT trial protocol (Hankonen et al., 2016). In brief, the intervention included two main strategies targeting motivation and capability – a poster campaign and a group programme with 6 small-group sessions – and two main strategies targeting opportunity – classroom choice architecture and teacher-led active classroom practices. For the first two strategies, central focus was on PA, while for the latter two, focus was on reducing SB (Figure 2).

Whereas PA is expected to change via a set of conscious motivational and self-regulation processes (see Supplementary Figure S3), changes in SB were designed to be introduced first and primarily by environment: changes in the physical choice architecture in classrooms (e.g. gym balls as chairs) and teacher-led changes (e.g. active classroom practices and activity breaks). In an intertwined way, the changed experience (due to the modified environment) would also later be accompanied with cognitive changes: Harms of excessive SB and tips for reducing SB in one’s daily life are presented in lessons, reinforced by poster campaign and personal experiences of less sitting.

However, due to overlapping and intertwining nature of PA and reduction of SB the group sessions also encouraged substituting sedentary activities with light PA (encouraging even small improvements).

As a key parameter for autonomous motivation, interaction principles based on SDT and (group) motivational interviewing were integral part of the group intervention. They included e.g. showing empathy for the students, positive feedback, providing structure and agenda, as well as options and choices (see full list in (Hankonen et al., 2016) and Supplementary Table S4). Making the activities in the group programme as participatory as possible and giving voice to the students was considered another key parameter for autonomous motivation.

We consolidated the principles and ideas into six Let’s Move It ‘theses’ or principles to make it clear and transparent what the programme endorses, and what possible misconceptions we are targeting (Supplementary Table S5). Finally, the final form of the intervention directed at students is described in the attached intervention description tables (Supplementary Tables S6 and S7). They include single behaviour change methods, tested in previous studies, to impact behaviour change, e.g. information about social norms, implementation intentions (Gollwitzer & Sheeran, 2006) and habit formation (Gardner, Lally, & Wardle, 2012). We used existing practical applications, e.g. the volitional help sheet (Armitage & Arden, 2010, 2012), and also we developed a set of our own individual exercises. As an example, a group task, named ‘Identifying Personal Motives Group Activity’, allowed sharing and learning from peers’ views on the benefits of PA and reflecting on their personal relevance. Another example was ‘Coping Plan Consultants’ where students, in small groups, discuss imaginary adolescents’ cases, to identify barriers to regular PA and collaboratively come up with solutions for these problems (coping planning/problem solving). These both exercises worked well and are described in detail in (Hankonen, Heino, Hynynen, et al., 2017) and Supplementary Table S6. Further discussion exercises target e.g. PA-related identity and self-perception, misconceptions of PA, as well as critical analysis of ‘fitspiration posters’ (see supplementary materials). Importantly, students were taught about motivation and behaviour change as well as how they themselves can play an active role therein (for a translated sheet from the workbook, see Supplementary Figure S4). All intervention materials are published in www.letsmoveit.fi

We took learnings from previous similar studies (see Hynynen et al., 2016). For example, Lubans and Sylva (2009) note that instead of intense interventions delivered by highly trained personnel in controlled settings, programmes should be realistic and situation specific, suitable for the school environment and designed to be implemented by teachers. Similarly, another trial evaluation (Mauriello et al., 2006) suggested intervention adjuncts such as a workbook or booster sessions to be useful in sustaining intervention effects. Thus, we incorporated student workbook and booster session in the final intervention, as well as a teacher manual for teachers.

## Discussion

This study described the phased development of a multi-level intervention programme, guided by the MRC framework on intervention development, the BCW as well as elements from the IM approach. The studies identified a range of relevant determinants to target in the intervention, in addition to identifying relevant BCTs. The final intervention was based mainly on SDT, self-regulation and control theories, with elements from the RAA, and consisted of two main parts, an individual-level intervention targeting autonomous motivation and imparting key self-regulatory skills that increased the capability of the student to increase PA, and a sitting reduction workshop intervention for their teachers, as well as other environmental changes. The activities and change methods were described in this paper and the extensive supplementary files.

This study is innovative in reporting the behavioural scientific development process in a detailed way, which is often called for but rarely done. The strengths of this project are (a) a sound basis on a systematic behavioural analysis synthesising previous research as well as qualitative and quantitative original research, (b) using systematically mapped, evidence-based strategies to influence behaviours (BCTs), including an explicit focus on also participants’ *enactment* of BCTs, (c) co-creation and pre-testing together with target group, (d) using also ‘best practices’ from the field, (e), targeting multiple levels of the system, (f) early on involving stakeholders and policy makers regarding the future dissemination and implementation and (g) conducting a step-wise design and development.

The process highlighted the relevance of behaviour change expertise. We recommend active hands-on work and being explicit about the principles of behaviour change with the advertisement and marketing professionals when producing intervention materials, so as to ensure the realisation of behavioural science principles in a most optimal way. It is common knowledge that to create implementable, acceptable and effective interventions, participant engagement in development is vital. However, the potential pitfall is that target groups are usually not experts in behaviour change – therefore, important decisions should not fully be left to the target group. For example, voting about the programme name can be left to targets, whereas BCT selection and combination should be informed by behaviour change science expertise. Yet, it should be noted that the LMI leaves quite a bit of tailoring and ‘personalisation’ within the intervention (as opposed to rigid non-adaptive intervention content or the other extreme, fully participatory development in each local context). For example, for the students, concrete PA plans are personalised to each individual and location, and the school staff are encouraged to select suitable activities out of a large menu of options, able thereby to decide the best choice for their context and situation, to realise the programme goal.

Studies informing the development were all designed to be fit for purpose, and fed into the intervention. Intervention development necessitates creativity, to come up with engaging and enjoyable ways to deliver effective BCTs in synergistic combinations, with e.g. gamification elements in face-to-face group setting. With several people immersed in the topic of youth PA and SB, unplanned innovations occurred – as an example, a sociological theory discovered during the write-up of the writing competition narratives was used as a core of a discussion exercise targeting rigid non-PA identities in the student sessions.

We have shed light on how intervention development necessarily involves painful decisions, as not all determinants can be targeted effectively nor can all potentially effective strategies be used due to resources or real-world constraints. It was useful to apply decision-making criteria across different steps, including considerations about intervention features. For example, balancing between effective features (e.g. higher dose and specific intervention content) vs. costs (affordability).

Intervention fidelity is a key aspect of interventions but often overlooked (Toomey et al., 2020). We considered different aspects of fidelity during the development of this intervention, and ways to ensure fidelity. Too often, fidelity is only conceptualised as intervention *delivery* (Toomey et al., 2020). An innovative part of the Let’s Move It development process was the attempt to consider the *enactment* aspect in detail: For example, we identified BCTs with sub-optimal enactment during the feasibility study (Hankonen, Heino, Hynynen, et al., 2017), and outlined expected BCT enactment from the perspective of participants (Supplementary Figure S2) to help identify possibly too high burden on the participants. We recommend developers to consider what the key self-enactable BCTs are that participants of universal interventions are prompted to enact, and when and how often. This may help improve ways to ensure enactment fidelity, as well as purposefully assess it.

There are several limitations. Optimally, the project would have allowed more time to optimise the intervention to support PA maintenance, and more systematic ways of incorporating injury prevention components. After the feasibility study and its 6 month follow-up, there was only 3 months until the start of the actual definitive trial, and with changes in staff, at this stage we were aware that time resources to produce components to promote longer-term maintenance were suboptimal. Also, the student intervention was delivered by research staff, even though optimally, more time would have allowed producing an additional intervention to train teachers to deliver the intervention and also activities to truly promote and support sustainable organisational change (and created a true effectiveness trial), but this was not possible given project funding constraints.

It should also be noted that despite the theories informing the intervention suggest dynamical, complex processes, the simplified pictorial representation of the intervention theory reduces these to static, linear and one-way relationships (see e.g. Rogers, 2008). As a limitation, we did not include explicitly possible emergent processes or draw feedback loops. What is necessary simplification in terms of clarity and communication, and what is too much, in terms of losing sight of important complexities that need to be captured accurately? Future work will hopefully enable ways to create of programme theories that will capture also more complex aspects of interventions (Rogers, 2008).

It should be noted that the evidence statements collated (Table 2) are simply a selection and not a comprehensive list of all learned content. Often during the intervention development process, evidence statements were implicitly considered (based on the studies), but they were not always formulated as explicit sentences or summaries. Furthermore, the team wrote internal reports and memos, some of which were in Finnish. In hindsight, more transparent reporting and thinking at each stage would have been welcome. We recommend that other intervention developers in the future publish their memos and interim reports (in line with recommendations, e.g. Bartholomew Eldrigde et al., 2016), and also consider doing this during the process openly, for example in the Open Science Framework, and collate key learnings regularly, during completion of different steps. This will help make the learning and progress explicit not only to the development team, but also to outsiders, who could even be invited to provide timely feedback to contribute to intervention development.

The study has several strengths: It demonstrates that it is possible to use a systematic and evidenced-based approach to intervention development and stakeholder co-creation integrating key principles from IM and BCW. Drawing from the strengths of each intervention development framework, we followed a more parsimonious intervention development process in line with the funding time and resources constraints. The frameworks and theories were a necessary backbone for the development, although adjustments and iterations needed to be made. The initial form of BCW in 2013 was complemented well by the IM’s detailed tips for various aspects of the development process.

## Conclusions

Interventions to target those from a lower educated background should be systematically developed, with good pre-testing, to target this specific group’s needs and contextual constraints, prior to a full trial. To our knowledge, this is the first time that the process of intervention development, for such a target sample, is systematically described alongside its feasibility test and further intervention refinement. This paper demonstrates how a systematic evidence synthesis, original research, theory and empirical optimisation informed the development and co-creation with stakeholder engagement of a complex intervention. We transparently show intervention content in detail, linking hypothesised behavioural determinants to the change techniques, and the innovative new group exercises that had high acceptability among vocational school youth.

## Supplementary Material

Supplemental Material
