# Supplementary material for: Changing activity behaviours in vocational school students: the stepwise development and optimised content of the ‘let’s move it’ intervention
Source: Health Psychol Behav Med. 2020 Sep 27;8(1):440–60. doi: 10.1080/21642850.2020.1813036 (PMC8114352; doi:10.1080/21642850.2020.1813036)

Supplementary Figure S2.

Let’s Move It student intervention development: Outlining expected BCT enactment over the course of the intervention.


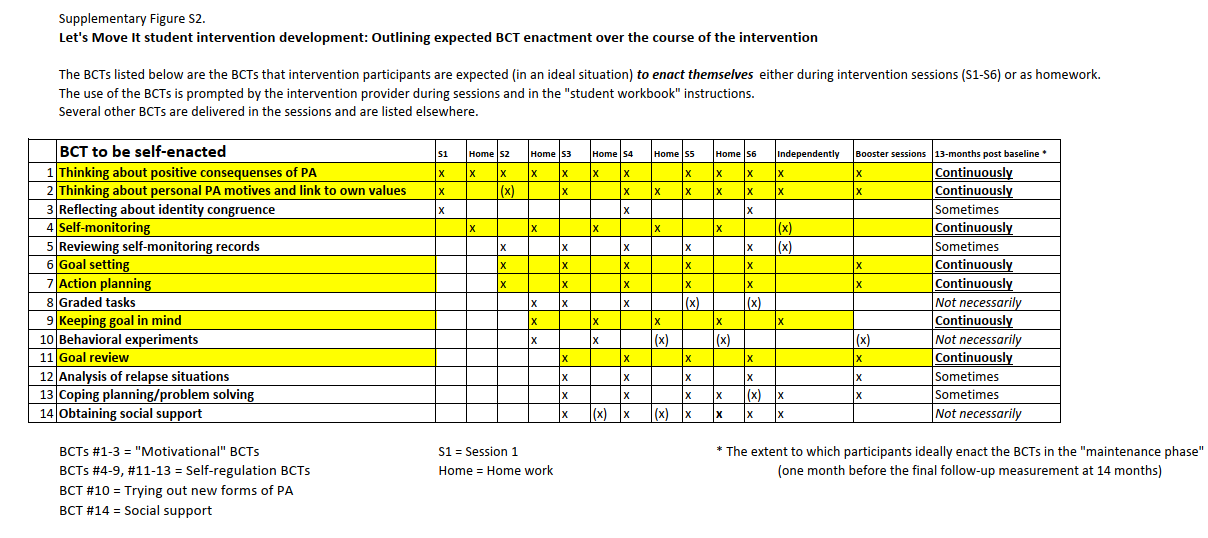

Supplement: Supplemental Material [file RHPB_A_1813036_SM8281.zip › suppl_data/S_Figure_S2_BCT_enactment_expectations_tool_used_in_development_phase_WORD (2).docx]
