# Supplementary material for: Changing activity behaviours in vocational school students: the stepwise development and optimised content of the ‘let’s move it’ intervention
Source: Health Psychol Behav Med. 2020 Sep 27;8(1):440–60. doi: 10.1080/21642850.2020.1813036 (PMC8114352; doi:10.1080/21642850.2020.1813036)
Supplement: Supplemental Material [file RHPB_A_1813036_SM8281.zip › suppl_data/S_Figure_S3_Simplified_picture_of_determinants_and_assumed_causal_logic_of_change-.docx]

**Supplementary figure S3. Simplified picture of determinants and assumed causal logic of change, depicting key determinants measured in questionnaires.**

**
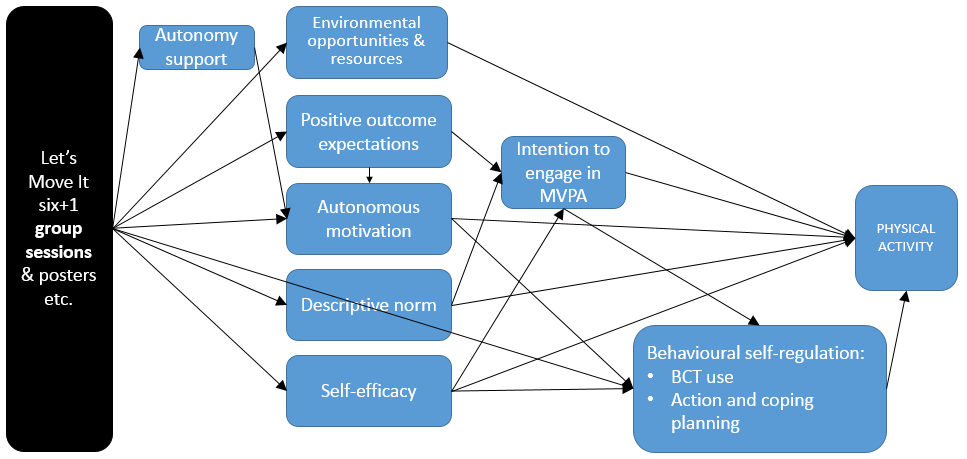
**
