# Supplementary material for: Changing activity behaviours in vocational school students: the stepwise development and optimised content of the ‘let’s move it’ intervention
Source: Health Psychol Behav Med. 2020 Sep 27;8(1):440–60. doi: 10.1080/21642850.2020.1813036 (PMC8114352; doi:10.1080/21642850.2020.1813036)
Supplement: Supplemental Material [file RHPB_A_1813036_SM8281.zip › suppl_data/S_Figure_S4_Lets_Move_It_toolbox_for_behavior_change-.docx]

**Supplementary figure S4. Let's Move It toolbox for behaviour change**

**GETTING THE HANG OF BEHAVIOR CHANGE**

This graph depicts some thoughts and ways which often affect whether or not the desired change is achieved. This “toolbox” can be utilized in other types of situations, too: in changing eating habits, controlling job application processes or something else. By pondering on these aspects, you are able to pinpoint where the issues might be and adjust your behaviour accordingly!


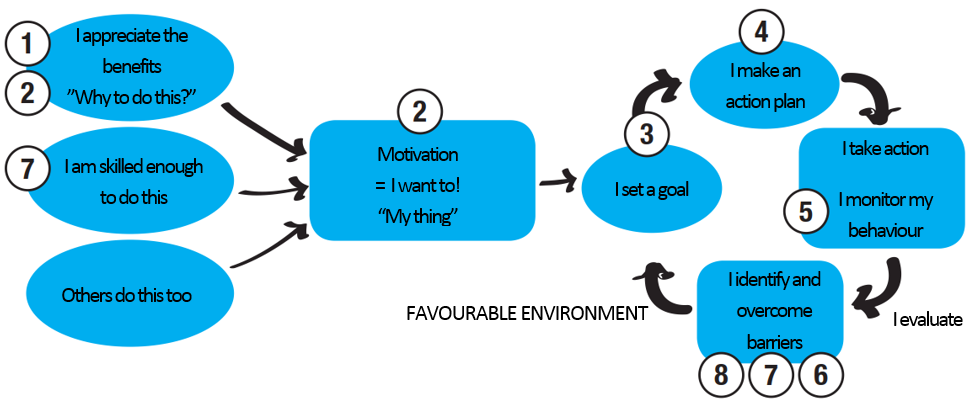


**LET’S MOVE IT TOOLBOX**

1. **Think about what are the important reasons FOR YOU to be physically active**You might not want to do 10 chin-ups and do a split. Think about the consequences that are important to you. Sleeping better? Being more creative? Coping with stress?
2. **Combine the things that are important to you or your values with physical activity.**For example, **those who love music** might enjoy dancing or running wearing headphones, you can go to the gym with a friend, different ball games are fun in a **group**, and **those close to nature** can opt for non-polluting muscle-powered forms of transportation or enjoy trips to forests. **Find the ways to be physically active that fulfil your values!**
3. **If you have concluded that you want to be physically active, specify a little.**

**SMART goal** is specific and measurable. Don’t be unspecific! Also, ensure that your goal is achievable and important to you!

1. **A detailed action plan increases the likelihood to be physically active.**A PA goal is more easily reached when you plan when, where and how to be physically active!
2. **Stay on the ball.**From time to time it is beneficial to have a PA diary or use sports app – this type of **self-monitoring** spurs physical activity.
3. **Know your barriers! Overcome them beforehand by planning solutions that suit you.**Someone diverts from their plans due to bad weather, another one gets stood up by a friend. When you know what kind of barriers you might encounter, you can plan for ways to overcome them and get closer to achieving your goals.
4. **Beware of discouraging beliefs.**Hopefully you don’t think that only exercising within an inch of one's life is beneficial? Or that physical activity is only for losing weight? Or that you should wait for inspiration or motivation before acting? Or one relapse erases all the previous efforts? No way! All in moderation, weight is not the main thing, acting generates motivation, and everyone is allowed to take it easy every now and then.
5. **Make the behaviour easy: Modify your environment.**When you set your sneakers in front of the door, pack your gym sack, prepare snacks beforehand, going for a PA session is easier – the ways are many!
