# Supplementary material for: Changing activity behaviours in vocational school students: the stepwise development and optimised content of the ‘let’s move it’ intervention
Source: Health Psychol Behav Med. 2020 Sep 27;8(1):440–60. doi: 10.1080/21642850.2020.1813036 (PMC8114352; doi:10.1080/21642850.2020.1813036)
Supplement: Supplemental Material [file RHPB_A_1813036_SM8281.zip › suppl_data/S_Table_S1_Studies_informing_LMI_intervention_development_FINAL_corrected-.docx]

**Supplementary table S1.** Evidence supporting intervention development conducted by the research group (eleven research articles described).

| Title, year, DOI link | Aims | Methods | Sample | Results |
| --- | --- | --- | --- | --- |
| Exercising and/or hanging out? Physical activity and friends in the lives of adolescents in vocational schools. (2013) *Liikunta & Tiede [Exercise & Science]* 50 (6), 32–39.  <https://fl-cdn.scdn1.secure.raxcdn.com/files/sites/4708/l-t-6-13_tutkimusartikkelit_liimakka_lowres-b0c2a661-092b-4c34-9d07-062bc07cc158.pdf> | PA relationship among this target group (Liimakka, Jallinoja, & Hankonen, 2013) | Individual interviews, qualitative analyses, interpretative phenomenological analysis | 15 vocational school students (both girls and boys) in different educational tracks | Around half of Finnish adolescents are insufficiently physically active. Students in vocational schools are less active than those in high schools. Using qualitative methods, this study investigated experiences, beliefs, and practices related to physical activity (PA) among youth in their first year of study in vocational schools. 15 adolescents were interviewed. Interpretative phenomenological analysis was used to analyse the data. Adolescents were categorized based on: leisure time choices; valuation of these; modes, forms and goals of PA. This lead to the identification of three groups with different PA relationships: target-oriented sports club youth, independent exercisers and hanging around. Many of the adolescents described a discrepancy, reflection, or time scheduling choice between hanging out with friends and PA (or exercise). The adolescents solved and experienced the dilemma between exercise and hanging out with their friends in different ways, and these solutions, in turn, were related to their PA relationship. The study offers implications for intervention design. Physical activity promotion might require, at least partly, different approaches for members of the three PA relationship categories identified. For example the “pull” of hanging out with friends, i.e., importance of peer group and the preferences for leisure-time are important especially in the “hanging out” category. |
| Autonomy support as basis of interventions to promote physical activity among children and adolescents. A literature review. (2015) *Kasvatus [Finnish Journal of Education], 46 (5), 473-89*.  <http://elektra.helsinki.fi/se/k/0022-927-x/46/5/autonomi.pdf> | To examine existing evidence and suitability of self-determination theory in promoting PA among youth | Literature review | Research articles on SDT-based studies in physical activity in children and youth | This narrative review examined the applicability of autonomy theory in explaining and enhancing children's and adolescents’ PA. Self-Determination Theory (SDT, Deci & Ryan 2000) in the context of youth schooling has shown that autonomy-based interaction can promote autonomous pupils' self-motivation, which is found to be associated with physical activity in both school and leisure time. The review presents the basic principles of self-determination theory and illustrates how theory based interventions can equip teachers and other practitioners working with health behaviour and change can with the necessary skills to support the regulation and motivation of autonomous behaviour of their students or clients. At the end of the review, skills used to support creating an autonomy supportive learning environment in physical education are discussed as well as the extent to which such interaction tools can be taught to counsellors. |
| Title, year, DOI link | **Aims** | **Methods** | **Sample** | **Results** |
| Osallistavalla otteella nuoret kumppaneiksi terveyden edistämisen tutkimuksessa. *Liikunta & Tiede*; 6, 8-15. | To review ways to conduct participatory intervention development/research. | Narrative review. | International research literature | Participatory approach has a positive impact on intervention outcomes. Broad participation can be achieved by offering adolescents enough opportunities for participation at different levels and in ways that meet their needs. Quantity and type of opportunities can be maximised by engaging adolescents in as many research phases as possible, by using a variety of methods for engagement, and by ensuring that the methods allow participation with different levels of commitment. |
| A systematic review of school-based interventions targeting physical activity and sedentary behaviour among older adolescents. (2016) *International Review of Sport and Exercise Psychology.* DOI:10.1080/1750984X.2015.1081706  <https://www.tandfonline.com/doi/abs/10.1080/1750984X.2015.1081706> | To evaluate the effectiveness of school-based interventions to increase PA and decrease SB among 15–19-year-old adolescents, and examine whether intervention characteristics (length, delivery mode and provider) and intervention content (i.e. BCTs) are related to effectiveness. | Systematic Review.  Aims and methods were prospectively registered in PROSPERO (<https://www.crd.york.ac.uk/prospero/display_record.php?RecordID=5531&VersionID=22752> ) | 10 cluster RCTs with measures of PA and SB were included, based on a systematic search. | 7/10 studies reported sig. increases in PA. Effects were small and short-term (Cohen's d 0.132- 0.659). 2/4 studies that measured SB reported significant reductions in SB. Interventions that increased PA included a higher number of BCTs, specific BCTs (e.g., goal setting, action planning and self-monitoring), and were delivered by research staff. Intervention length and mode of delivery were unrelated to effectiveness. |
| Relations Between Autonomous Motivation and Leisure-Time Physical Activity Participation: The Mediating Role of Self-Regulation Techniques. *Journal of Sport and Exercise Psychology* 38(2):128-37.  <https://journals.humankinetics.com/doi/10.1123/jsep.2015-0222> | To test the predictive validity of a multi theory model analysing the effect of autonomous motivation (from self-determination theory) on physical activity participation and testing mediation by self-regulatory techniques (from control theory). | Prospective survey including validated measures of the predictors and physical activity, at baseline and after one month.  A subsample used an accelerometer to objectively measure physical activity and further validate the physical activity self-report assessment tool (n = 44). | Finnish adolescents (*N* = 411, aged 17–19) completed baseline and at one month (N=177). | Autonomous motivation statistically significantly predicted action planning, coping planning, and self-monitoring. Coping planning and self-monitoring mediated the effect of autonomous motivation on physical activity, although self-monitoring was the most prominent. Controlled motivation had no effect on self-regulation techniques or physical activity. *Developing interventions that support autonomous motivation for physical activity may foster increased engagement in self-regulation techniques and positively affect physical activity behaviour.* |
| Title, year, DOI link | Aims | Methods | Sample | Results |
| Acceptability of Strategies to Reduce Student Sitting: A Mixed-Methods Study With College Teachers. *Health Promotion Practice*. DOI: 10.1177/1524839916677209  <https://journals.sagepub.com/doi/abs/10.1177/1524839916677209> | Teachers are key agents to deliver interventions to reduce youth sedentary behaviour in school setting (where students spend most of their time). We aimed to examine teachers’ current use and willingness to use various strategies to decrease student sitting and potential barriers and facilitators of its use in a future intervention. | Mixed-methods design with college teachers using an online cross-sectional survey and focus group interviews. | Survey:  *n* = 192.  Focus group interviews: *n* = 13. | Although a vast majority (87%) of the teachers found reducing prolonged sitting an important goal, only 47% were actually including practices to reduce sitting in their classroom. 89% of the teachers reported willingness to use at least one of the five alternative strategies presented. Focus groups revealed a discussion emphasis on environmental opportunity and motivation as key to implementation. Teachers also generated additional ideas for intervention content.  Despite low levels of current sitting reduction, teachers were willing to try at least one strategy to reduce sitting. Results informed intervention development regarding parameters of use for each strategy*. When possible, interventions should provide teachers with a variety of alternative strategies that are easy to use to reduce prolonged sitting.* |
| From play to hobby, benefit and games: Meanings of physical activity and its change in competitive writings of adolescents. *Psykologi*a 52 (1).  <http://www.psykologia.fi/arkisto/vanhat-numerot/vanhat-numerot-ghost/471-2017-01/tieteelliset-artikkelit/597-leikista-harrastukseen-hyotyyn-ja-pelailuun-liikunnan-merkitysten-muutokset-nuorten-kilpakirjoituksissa> | To investigate youth perceptions of why they increase their activity. While many previous qualitative studies of change have adopted a focus on maintenance or dropping out of regular PA, this study investigated interpretations of becoming physically active. | Narrative analysis, frame analysis | Writings (n=115) of 15–24-year-old adolescents, acquired in a writing contest from both vocational and high schools | The adolescents interpreted the increase in their activity by using four different frames that described physical activity as 1) play, 2) hobby, 3) personal benefit and 4) hanging around with friends. The relevant distinction-makings related to becoming physically active were combined in the narratives to the changes in or diversification of frames. Over the lifespan, narratives of physical activity as play and as games during breaks first changed into descriptions of physically active hobbies. Later, the hobby frame was joined by novel interpretations. In terms of becoming active, immediate benefits gained via physical activity were central, for example, feeling good and more energetic. Positive experiences from physical activity and a personal insight of the association between physical activity and one’s own well-being were related to intentions to continue physical activity also in the future. Based on the results, physical activity interventions should also emphasize benefits for daily life, in addition to long-term health consequences. |
| Title, year, DOI link | Aims | Methods | Sample | Results |
| What triggers changes in adolescents’ physical activity? Analysis of critical incidents during childhood and youth in student narratives  Preprint DOI:10.31234/osf.io/fk5tx, address: <https://psyarxiv.com/fk5tx/> | To understand dynamic PA change by identifying narrative triggers that adolescents themselves relate to their PA change | A qualitative and inductive approach, Critical Incident Technique (CIT) | Writings (n=115) of 15–24-year-old adolescents, acquired in a writing contest from both vocational and high schools | Seven critical incident categories were identified: 1) promoting one’s own well-being, 2) encountering health problems, 3) becoming aware of body image ideals, 4) finding an inspiring sport or losing the inspiration of sport, 5) experiencing transitions in life circumstances, 6) receiving support or lack-of-support from significant others, and 7) becoming adult. The first three were described as triggers of agentic PA increase in adolescents’ stories. |
| A Dual Process Model to predict Adolescents’ Screen Time and Physical Activity: Reasoned Action Approach and Automaticity (in review) | To identify salient behavioural beliefs among Finnish adolescents, and  to evaluate to what extent reflective processes (the Reasoned Action Approach) and behavioural automaticity predict screen time and PA | A belief elicitation study. Prospective survey, structural equation modelling. | Belief elicitation study: n= 44.  Survey: Adolescents (*N* =411, mean age 17.8 years) responded to a survey, with a follow-up after one month.  PA, accelerometry, sub-sample (*N*=44). | The most frequent salient outcome beliefs regarding screentime were muscle stiffness (disadvantage) as well as gaining information about the world and communication with people (advantage). Screen time and PA were predicted by intention and past behaviour. Automaticity predicted weekday screen time directly but weekend screen time and PA indirectly via intention. Outcome expectancies, self-efficacy, automaticity and past behaviour predicted intentions for screen time and PA. |
| Title, year, DOI link | Aims | Methods | Sample | Results |
| What explains the Socioeconomic Status Gap in Activity? Educational Differences in Determinants of Physical Activity and Screentime. *BMC Public Health,* 17:144. DOI: 10.1186/s12889-016-3880-5  <https://bmcpublichealth.biomedcentral.com/articles/10.1186/s12889-016-3880-5> | We aimed to investigate a comprehensive range of potential theoretical mediators of physical activity (PA) and screen time in different socioeconomic status (SES) groups: a high SES group of high school students, and a low SES group of vocational school students. The Theoretical Domains Framework (TDF), was used as an heuristic framework to synthesise different theoretical determinants in this exploratory study mapped onto to COM-B. | Survey of Finnish vocational and high school students (*N* = 659) aged 16–19. Responded to psychological, social and environmental determinants of activity (PA and screen time). The outcome measures were validated self-report measures for PA and screen time. | 659 vocational students. | For PA, there were SES differences in all of the COM-B domains. Vocational school students reported using less self-monitoring of PA, weaker injunctive norms to engage in regular PA, and fewer intentions than high school students. Mediation analyses identified potential mediators of the SES-PA relationship in all of three domains: self-monitoring (CI95 for b: 0.19–0.47), identity (0.04–0.25) and material resources available (0.01–0.16). SES was not related to most determinants of screentime, where there were mainly gender differences. Most determinants were similarly related with both behaviours in both SES groups, indicating no major moderation effect of SES on these relationships. |
| Title, year, DOI link | Aims | Methods | Sample | Results |
| Randomised controlled feasibility study of a school-based multi-level intervention to increase physical activity and decrease sedentary behaviour among older adolescents. *International Journal of Behavioral Nutrition and Physical Activity,* 14: 37, doi: 10.1186/s12966-017-0484-0  <https://ijbnpa.biomedcentral.com/articles/10.1186/s12966-017-0484-0> | This study investigates feasibility and acceptability of two main intervention components and research procedures, and uptake of behaviour change techniques (BCTs) by the participants. | Design was an outcome assessor blinded, cluster-randomised controlled trial. The intervention consisted of (1) a 6-h group-based intervention for students, (2) two 2-h training workshops to reduce their students’ sitting in class for teachers, and (3) provision of light PA equipment in classrooms. At baseline (T1), mid-intervention (T2) at 3 weeks, post-intervention (T3) and 6 months after baseline (T4) we measured hypothesised psychosocial mediators and self-reported PA and sitting. Objective assessment of PA and SB (7-day accelerometry) was conducted at T1, T3 and T4.Students and teachers in the intervention arm filled in acceptability questionnaires at T3. | Four classes of students (matched pairs) were randomised into one intervention and one control arm. | Recruitment rate was 64% (students) and 88.9% (teachers), and at T3, all post-intervention measurements were completed by 33 students (retention 76.7%) and 15 teachers (retention 93.8%). Acceptability ratings of sessions were high (students M = 6.29, scale 1–7), and data collection procedures were feasible. Intervention arm students reported increased use of BCTs, but uptake of some key BCTs was suboptimal. BCT use correlated highly with objective measures of PA. Based on both self-report and student evaluation, teachers in the intervention arm increased the use of sitting reduction strategies at post-intervention and T4 follow-up (p < .05).  We detected willingness of the target groups to participate, good response rates to questionnaires, adequate retention, as well as acceptability of the trial protocol. Investigation of BCT use among students helped further enhance intervention procedures to promote BCT use. After making necessary modifications identified, intervention effectiveness can next be tested in a definitive trial. |
