# Supplementary material for: Changing activity behaviours in vocational school students: the stepwise development and optimised content of the ‘let’s move it’ intervention
Source: Health Psychol Behav Med. 2020 Sep 27;8(1):440–60. doi: 10.1080/21642850.2020.1813036 (PMC8114352; doi:10.1080/21642850.2020.1813036)
Supplement: Supplemental Material [file RHPB_A_1813036_SM8281.zip › suppl_data/S_Table_S2_Overview_of_effective_elements_of_school_based_health_promotion-.docx]

**Supplementary table S2.** Overview of effective elements of school-based health promotion.

| **The Let’s Move It intervention development team aimed to use these principles:** |
| --- |
| - Design carefully   - Use theory, carefully define target behaviours and target groups     - Focus on PA (not multiple health behaviours as target)   - Engage the target group in intervention design, takes an interest in special needs |
| - Use several components to target behaviour   - Do not resort to classroom-based education alone     - Attend to social influences   - Target motivation and cognitive-behavioural skills (e.g., goal setting, planning) |
| - Ensure intervention fidelity   - Carefully educate implementers and provides continual support |
| - Form partnerships with actors outside the school |

Based on reviews by *Kreimler et al. 2011, Dadaczynski & De Vries 2013, Peters et al 2010).*
