# Supplementary material for: Changing activity behaviours in vocational school students: the stepwise development and optimised content of the ‘let’s move it’ intervention
Source: Health Psychol Behav Med. 2020 Sep 27;8(1):440–60. doi: 10.1080/21642850.2020.1813036 (PMC8114352; doi:10.1080/21642850.2020.1813036)
Supplement: Supplemental Material [file RHPB_A_1813036_SM8281.zip › suppl_data/S_Table_S3_The_main_principles_of_designing_the_Lets_Move_It_poster_campaign-.docx]

**Supplementary Table S3. The main principles of designing the Let’s Move It poster campaign**

| Continuous collaboration between the research team and the advertisement agency ensured that the following core principles derived from the theories and practical insights were followed in all materials:   - Joy, positive feelings, humour, not too serious - Avoid control and coercion - Emphasize autonomy and voluntariness - Based especially on autonomous and intrinsic goals - Provide rationales: knowledge and understanding are essential for autonomous motivation - Avoid exaggeration of benefits and creation of unrealistic outcome expectations - Support of sense of competence and self-efficacy - Inclusiveness: messages targeted directly at the low active youth - Satisfaction of behaviour and its outcomes/consequences are essential when adopting a new behaviour and maintaining the behaviour change - focus on successful experiences. Also drawing attention to positive outcomes to enhance the satisfaction - Modelling (narrative stories of same-aged adolescents) are used in order to highlight the meaning of personal experiences, and to promote self-efficacy/competence - Emphasis on short-term benefits of increasing PA, framed positively, emphasizing intrinsic goals - Positive feedback about the new behaviour; especially reflecting the discrepancy between current behaviour and the person’s previously set goal is useful   Link to posters: <https://drive.google.com/open?id=18DwvbXj5KfPRWfXDfbwvwbZfDszNA3TW> |
| --- |
