# Supplementary material for: Changing activity behaviours in vocational school students: the stepwise development and optimised content of the ‘let’s move it’ intervention
Source: Health Psychol Behav Med. 2020 Sep 27;8(1):440–60. doi: 10.1080/21642850.2020.1813036 (PMC8114352; doi:10.1080/21642850.2020.1813036)
Supplement: Supplemental Material [file RHPB_A_1813036_SM8281.zip › suppl_data/S_Table_S4_Interaction_principles-.docx]

**Supplementary table S4. Interaction principles (originally published in Hankonen et al., 2016)**

| 1. Show empathy for students 2. Ask open questions 3. Roll with students’ resistance 4. Evoke change talk 5. Show interest in students’ experience and perspectives 6. Provide students with options and choices 7. Provide students with structure and agenda 8. Use reflective listening 9. Validate students´ concerns 10. Provide positive feedback |
| --- |
