# Supplementary material for: Changing activity behaviours in vocational school students: the stepwise development and optimised content of the ‘let’s move it’ intervention
Source: Health Psychol Behav Med. 2020 Sep 27;8(1):440–60. doi: 10.1080/21642850.2020.1813036 (PMC8114352; doi:10.1080/21642850.2020.1813036)
Supplement: Supplemental Material [file RHPB_A_1813036_SM8281.zip › suppl_data/S_Table_S5_Lets_Move_It_principles_theses_-.docx]

**Table S5. Let's Move It principles (theses)**

| **ANY MOVEMENT IS GOOD! ANY ACTIVITY IS BETTER THAN NOTHING**  The black-and-white thinking that physical activity should be “all or nothing” is dysfunctional, one does not have to reach recommended levels in order to benefit from activity. Every little bit counts, even small increases to PA bring various health benefits**.** Taking baby steps (“graded tasks”) is useful. Building a sense of competence and self-efficacy.  **YOUR OWN CHOICE: WHETHER YOU ARE ACTIVE, AND HOW.** It is everyone’s personal choice whether or not they are active, and the LMI does not force anyone to do more PA. Supporting the need for autonomy.  **WE ARE ALL ENTITLED TO ACTIVITY.** Emphasizing opportunities, emphasizing that you do not need to be an ‘athlete’ or of a certain body shape or size to enjoy PA.  **KNOW WHAT MOVES YOU.** We all do not have to like all forms of activity. It is helpful to identify one’s key personal motives for PA. How can PA support what is valuable for you in life? LMI explicitly supports the formation of intrinsic goals (in line with goal content theory of the SDT).  **GOAL IS WELL-BEING, NOT FATLESS BODY**. A usual misconception is that PA is mostly about getting lean and good-looking. These goal motivations are not explicitly endorsed by the LMI due to their potential inefficacy and side-effects, and instead, more intrinsic goals are suggested.  **SITTING SUCKS.** Rest is important, but in our society sitting has been made “unnaturally” easy. Reducing excessive sitting and taking even small breaks are good for the body and mind! Sitting is a health risk independent of levels of PA. |
| --- |
