# Supplementary material for: Changing activity behaviours in vocational school students: the stepwise development and optimised content of the ‘let’s move it’ intervention
Source: Health Psychol Behav Med. 2020 Sep 27;8(1):440–60. doi: 10.1080/21642850.2020.1813036 (PMC8114352; doi:10.1080/21642850.2020.1813036)
Supplement: Supplemental Material [file RHPB_A_1813036_SM8281.zip › suppl_data/S_Table_S6_The_Lets_Move_It_Intervention_LMI_student_intervention_sessions_BCTs_and_determinants-.docx]

**Supplementary Table S6. The Let’s Move It Intervention (LMI), student intervention sessions (6 + booster): BCTs and determinants.**

| ***Exercise*** | ***Objectives*** | ***Activity description*** | ***Behaviour Change Techniques (BCTs) for PA and/or SB*** | ***Materials*** | ***Determinants of PA &/or SB*** |
| --- | --- | --- | --- | --- | --- |
| “START” | SESSION 1  (60 min) | **Summary:**   - **Introduction** - **Setting the group rules** - **Establishing rapport** - **Perceptions of physical activity, discussing misconceptions** - **Consequences of excessive sitting or sedentary time** - **Introduction to self-monitoring** |  |  |  |
| **Welcome**  **(2-5 min)** | Students:   - understand the rationale for the intervention, the LMI-program and its main targets and working methods; - increase the motivation to participate in LMI program; - develop a safe and positive climate in the classroom. | - Facilitator introduces him-/herself. Facilitator shortly presents the rationale for the LMI-program and outlines what will be discussed during the next 6 group sessions targeting PA and wellbeing. - Expectations for participation are explored. | N/A | PowerPoint-slides: Setting the stage | Positive, safe climate |
| **Warming up: Mood/feeling cards**  **(10 min)** | Students:   - introduce themselves to each other and the LMI facilitator; - get a feeling of being welcome and accepted; - increase psychological safety in the context to increase functional group interaction in later sessions | - For the introductions, each student is directed to pick a mood card from the table, representing his/her mood for the day. - Taking turns, each person shows the card to the group and introduces him/herself. - Facilitator participates, and begins the introduction round to demonstrate that sharing feelings (also negative) is allowed. | N/A | Mood cards | Positive, safe group climate |
| **Good group & group rules**  **(10 min)** | Students:   - define group norms and ground rules for working together, including interaction and confidentiality; - commit to the rules and take responsibility for the sessions; - will feel that the group climate is safe | - Facilitator asks questions (projected also onto the screen): “What is a good group? What kind of a group would you like to be a part of?” - Students discuss the group rules in self-selected pairs. - After pair discussions, each pair is asked to contribute at least one important rule for the | N/A | PowerPoint slides | Positive, safe group climate |
| ***Exercise*** | ***Objectives*** | ***Activity description*** | ***BCTs for PA and/or SB*** | ***Materials*** | ***Determinants of PA and/or SB*** |
| **(cont.)** | and that in these lessons positive interactions and confidentiality can be expected | - group and the facilitator lists these for the group on a flip-over sheet. - The facilitator emphasizes that there are no right or wrong rules and encourages every pair to take part in the discussion. - Facilitator makes sure that all rules are understood and approved by every group member before progressing. |  |  |  |
| **PA identity continuum**  **(10 min)** | Students:   - broaden their understanding of what PA entails (not just PE or vigorous exercise); - identify current levels and types of PA; - reflect on own PA identity and realize it can be viewed in more nuanced ways, including a continuum, compared to a simple dichotomy “active individuals vs. inactive individuals”; - identify ways in which they in fact already are active – even small bouts of activity count as PA (i.e. PA already is a part of their life), thus questioning identity as a passive person (Main message: We are all entitled to activity); - produce positive change talk about PA and their future goals; - understand the main targets of the LMI program   - Everyone does not have to become a sport enthusiast;   - Even a small enhancement in PA is better than nothing (gradual increases on the continuum); and,   - PA helps improve well-being | - Facilitator starts by showing a slide presenting a common but a very black-and-white, dichotomous view of PA identity (“People are either sporty or unsporty”). The facilitator then challenges the dichotomy with a more fine-grained, multiple identity model that distinguishes “guests”, “tourists”, “regular visitors” and “insiders” (as defined by Unruh^1^ (see also Table S6-b). The facilitator highlights the broad variety of PAs and explains how a person can be a guest in one form of PA while being an insider in another. - Facilitator asks about familiarity with different forms of PA. Facilitator emphasizes especially moderate or light forms of PA (e.g. house cleaning, taking stairs instead of using the lift). - Students discuss, first in pairs and then in the whole group, about where they situate themselves in the PA model. ”In which position are you in different forms of PA at the moment, and where would you perhaps want to be in the future?” - Facilitator emphasizes the benefits of even small changes and supports identification of existing forms of PA in students’ daily lives. - Facilitator leads discussion towards common understanding that daily activities such as stair climbing are PA. | 13.5. Identity associated with changed behaviour  15.1. Verbal persuasion about capability  5.1. Information about health consequences  15.3. Focus on past success | PowerPoint slides | Knowledge  Outcome expectations  Self-efficacy  Autonomous motivation (integrated regulation) |
| ***Exercise*** | ***Objectives*** | ***Activity description*** | ***BCTs for PA and/or SB*** | ***Materials*** | ***Determinants of PA and/or SB*** |
| **Yes, we can & Identifying Personal Motives Group Activity**  **(10 min) (see also Hankonen et al., 2017^3^)** | Students:   - understand that everyone is entitled to be physically active/exercise (e.g. no matter how they look during the exercise); - reflect on the most important personally meaningful reasons to be physically active; - learn about the various positive consequences of PA; - link physical activity to their current wellbeing concerns and values; - hear other students talk about PA and its consequences in a positive way; - recognize that moderate activity also benefits (it doesn`t have to be all or nothing); - engage in “change talk”, arguments in favour of PA - understand that it is possible to enhance/develop PA motivation. | - Facilitator shows the group This Girl Can Campaign video from YouTube. In this video ordinary young women with different body weights and shapes exercise and have fun with friends.^2^ - Students discuss with the whole group: “To whom does PA belong? Does it matter what you look like when you do PA, e.g. sweaty?” - Facilitator leads the discussion towards enjoyment and social aspects of PA, and emphasizes that one can learn to enjoy PA. - Facilitator places written cards on benefits of PA on the table. - Students pick up one or more benefit cards after thinking about the most important reasons they find for being physically active (or the most inspiring reason to begin to be more active). - Students show the benefit card they have chosen and talk about it to others. Facilitator makes sure that everyone gets their turn to participate. - Facilitator leads the discussion to identify a broad variety of positive consequences of PA. - Facilitator highlights that everyone has their own and personally meaningful reasons to be physically active (e.g. not everyone is motivated by competition) and that students can increase their motivation in multiple ways (if they want). - If students are reluctant to select a card or talk about it, this is accepted. | 5.1. Information about health consequences  5.2. Salience of consequences  5.3. Information about social and environmental consequences  5.4. Information about emotional consequences  6.3. Information about others’ approval  13.2 Framing/Reframing  6.3 Information about others’ approval  16.3 Vicarious consequences | This girl can video from YouTube  PA benefit cards | Descriptive norm  Autonomous motivation (integrated motivation regulation)  Outcome expectations  Knowledge  Self-efficacy |
| **Sitting statement & discussion: Sitting in our society?** | Students:   - become aware how much passive time they have during a normal weekday / weekends, and arrangement of our living environments; - learn negative consequences of sedentary lifestyle (personally and in general) and become more motivated to reduce excessive sitting; | - Facilitator projects a sitting statement on the wall “Our society drives us to live sitting down” (“Maailma on suunniteltu istuen elettäväksi”). - Facilitator asks the students to stand up and discuss the statement with a partner, encouraging both pro and con arguments - The pairs are asked to report back to the whole group: “Is the statement true? Why? Why not?“ | 5.1 Information about health consequences  5.2 Salience of consequences  5.3 Information about social and | PowerPoint slides | Descriptive norm    Autonomous motivation  Outcome expectations (also SB) |
| ***Exercise*** | ***Objectives*** | ***Activity description*** | ***BCTs for PA and/or SB*** | ***Materials*** | ***Determinants of PA and/or SB*** |
| **(cont.)** | - learn that the negative consequences follow both from sitting and lying down (total passive time); - get practical experience of sitting reduction and tips how to do it | - Facilitator directs the discussion to provide students with an opportunity to reflect upon their own sitting habits and to share their personal observations about excessive sitting time and its consequences. - The whole group discusses a poster in which negative consequences of excessive sitting time are listed. After that students share easy tips on how to decrease their own sitting time and incorporate sitting reduction into daily life. - Facilitator avoids “demonization” of sitting and highlights how easy and even fun sitting reduction could be. - Facilitator instructs students on how to use PA equipment and prompts students to use them at least once after the equipment has arrived in the classrooms. | environmental consequences  5.4 Information about emotional consequences  6.1 Demonstration of behaviour   8.1 Behavioural practice and rehearsal (SB) *(equipment guidance)*  4.1 Instruction on how to perform the behaviour (SB) *(tips & equipment guidance)*  6.3 Information about others’ approval  12.3 Restructuring the physical environment  16.3 Vicarious consequences |  | Knowledge (also SB) |
| **Weekly individual ACTION exercise (i.e., home assignment), PA self-monitoring diary  & wrap-up of the session** | Students:   - form an intention to self-monitor all forms of PA and feelings evoked by PA for the following week (recording daily exercises in a PA diary, as well as how they feel when engaging in PA). | - Students get personal copies of the LMI workbooks. - Facilitator gives an overview of the content and purpose of the workbook. - Facilitator instructs how to keep an individual PA and mood diary (in the workbook, or digitally, e.g. in a mobile app of their choice) and explains the benefits of the self-monitoring activity. | 2.3. Self-monitoring (behaviour)  5.6. Monitoring of emotional consequences  3.2. Social support (practical) | Workbooks (PA diary)  Information sheets to parents  Pencils | Behavioural self-regulation |
| ***Exercise*** | ***Objectives*** | ***Activity description*** | ***BCTs for PA and/or SB*** | ***Materials*** | ***Determinants of PA and/or SB*** |
| ***(cont.)*** |  | - Students get an information sheet to take home to their parents/custodians/other family members with practical examples on how they can support the youth’s PA pursuits, and advise them to refrain from negative interactions regarding PA (although well-intentioned), e.g. pressuring the youth to do PA.   Facilitator guides the students to summarize the most important topics of the week using open-ended questions (What is a good group? My PA identity? What are the positive consequences of PA? Sitting and its reduction?) | 7.5 Remove aversive stimulus | PowerPoint slides |  |
| “GET FIT” | SESSION 2  (45 min) | **Summary:**   - **The most important messages/principles of the LMI-program** - **Dimensions/components of physical fitness** - **What are the pros of light daily activity?** - **Goal setting** |  |  |  |
| **The most important messages of last session and main messages of Let’s Move It program**  **(5 min)** | Students:   - revisit the most important messages of session 1; - for those who did not attend session 1: understand the structure of Let’s Move it program; - internalize the main messages of the LMI-program; - understand what constitutes physical fitness and learn about PA recommendations; - learn the basics of goal setting by setting an easy, daily incidental activity goal for the next week. | - Facilitator guides a whole group discussion to revisit session 1 and its’ main messages (see S1 PA Identity Continuum and Identifying Personal Motives Group Activity). - Facilitator presents the group rules from session 1 (see S1 good group). - Facilitator presents the six main messages of the LMI- program - Adding any movement is good! - Your own choice: whether you are active, and how. - We are all entitled to activity. - Know what moves you. - Goal: well-being, not fatless body. - Sitting sucks. - Facilitator guides a discussion on the messages - How do you feel about the messages? - Do the messages sound reasonable? | 15.1 Verbal persuasion about capability  4.2 Information about antecedents of behaviour  5.1 Information about health consequences  5.6 Information about emotional consequences  5.3 Information about social and environmental consequences | PowerPoint slide | Positive, safe group climate  Self-efficacy  Knowledge  Outcome expectations |
| ***Exercise*** | ***Objectives*** | ***Activity description*** | ***BCTs for PA and/or SB*** | ***Materials*** | ***Determinants of PA and/or SB*** |
| **(cont.)** |  |  | 5.2 Salience of consequences  6.3 Information about others’ approval  16.3 Vicarious consequences  15.3 Focus on past success |  |  |
| **ACTION exercise review: PA diary**  **(15 min)** | Students:   - learn to do and review self-monitoring of the behaviour; - recognize ways to make their days a little more active; - share useful tips on how to remember to fill in the PA diary in the future. | - Facilitator presents the following questions for students to discuss the PA diary first in pairs and then with the whole group: - What kind of exercise did you do? - How did it feel to keep a PA diary? - If you had some problems (e.g. forgot) filling up the diary, how could you remember better next time? - What could you do to make your days a bit more active? - Every pair gives at least one answer. - After the discussions, students are invited to write their activity tips on the blackboard. | 2.2. Feedback on behaviour *(review of self-monitoring records)*  6.2. Social comparison  5.4. Monitoring of emotional consequences | PowerPoint slide  Workbook | Behavioural self-regulation  Descriptive norm  Outcome expectations |
| **Fitness knowledge quiz (2 parts) (20 min) (see also Hankonen et al., 20173)** | Students understand:   - that physical fitness consists of three different dimensions (strength, mobility and endurance); - what kind of sports enhance the different dimensions of physical fitness; - that some ways of exercising can improve all three (e.g. gymnastics) while others focus more on other areas (e.g. yoga on mobility & strength, or running on aerobic fitness) | - In a playful quiz, students answer questions related to physical fitness in groups of five (1st round: What kind of sports enhance the three different dimensions of physical fitness – come up with as many forms of PA as you can!; 2nd round: How often should adolescents exercise according to the recommendations?) - Students are given 2 minutes to write down their answers. Facilitator acts as the referee. - Facilitator highlights that it is a playful quiz and nothing too serious | 4.1. Instructions on how to perform a behaviour  5.1. Information about health consequences  5.2. Salience of consequences | PowerPoint slides  Quiz sheets (Dimensions of PA and Recommendations)  Pencils | Knowledge  Self-efficacy  Autonomous motivation  Outcome expectations |
| ***Exercise*** | ***Objectives*** | ***Activity description*** | ***BCTs for PA and/or SB*** | ***Materials*** | ***Determinants of PA and/or SB*** |
| **(cont.)** | - how often they should exercise according to the recommendations; - that it is important to start exercising with baby steps by learning the basics of injury prevention; - how to connect the different areas of physical fitness to outcomes they themselves value; - how to enhance their self-efficacy; - how and why to use LMI workout videos, also at home. | - After each round, small groups present their answers to the entire group, and the facilitator reveals correct answers by complementing the students’ accounts. Scores are written on the table. - Facilitator highlights that the PA recommendations may not be applicable to each individual and their situation, especially if you haven’t been physically active before (every step counts, and every movement is better than nothing). - Facilitator explains how different dimensions of PA should be trained equally, always considering the starting level (rules for safe training). - When wrapping up after the quiz, facilitator highlights the importance of finding “your own thing”. Only students themselves can be aware of their personal PA motives and link PA to their own values (e.g. they can integrate PA to hanging out with a friend). - Finally, facilitator presents the LMI home workout videos, that have been titled according to the dimensions of fitness that they help to improve | 8.7. Graded tasks *(focus on starting with small steps)*  15.1. Verbal persuasion about capability | Blackboard |  |
| **ACTION exercise: Incidental activity**  **& wrap-up of the session**  **(5 min)** | Students:   - learn the basics of goal setting by setting an easy “Daily incidental activity”-goal for the next week; - form an intention of how the goal is realised, and increase daily PA over the next week; - are motivated to monitor how they felt after pursuing the Daily incidental activity. | - Students familiarize themselves with the Daily incidental activity checklist in their workbooks. Here, daily activity checklist provides easy ways to integrate breaks in SB or light PA in one’s daily life, e.g. standing up in the bus. - Students pick up and mark one or more ways which they feel inspiring or easy enough to pursue during the next week. - During goal setting, the facilitator asks the students to think of important personal reasons for being more active (facilitator avoids coercion and supports students’ intrinsic motivation). | 1.1. Goal setting (behaviour)  8.1. Behavioural practice/rehearsal  8.2 Behaviour substitution  1.4. Action planning  8.7. Graded tasks  2.3. Self-monitoring of behaviour | Workbook    Pencils | Behavioural self-regulation  Autonomous motivation |
| ***Exercise*** | ***Objectives*** | ***Activity description*** | ***BCTs for PA and/or SB*** | ***Materials*** | ***Determinants of PA and/or SB*** |
|  |  | - To wrap up the session, the facilitator asks questions about the most important topics of the session and lets the students summarize (“Physical fitness consists of three different dimensions. What are these dimensions? What are the pros of daily incidental activity?”). | 5.4. Monitoring of emotional consequences *(prompted in the PA diary)*  8.3. Habit formation *(only SB)* |  |  |
| “HOW AND WHERE?” | SESSION 3  (45 min) | **Summary**   - **Review of behaviour goal(s)** - **PA experiment** |  |  |  |
| **The most important messages of session 2: How fit am I? How can I test my physical fitness level?**  **(10 min)** | Students:   - recall the basic knowledge of the dimensions of physical fitness and recall how they can enhance their physical fitness; - get an idea of their current level of physical fitness (endurance, strength, mobility) by participating in physical exercise; - experience the instant positive consequences of PA. | - Students test their own PA level with three functional and playful exercises and at the same time, go through the previous week’s PA fitness knowledge (strength: squats, as many reps as possible in 60 s./ endurance – taking stairs (3 floors) as quickly as possible and talking at the same time / mobility – forward stretch). - Facilitator gives instructions on how to perform the movements and facilitator also participates in the test. - Facilitator highlights that the test is meant to be playful and participation is voluntary (avoid coercion). - Facilitator asks how students feel after the test and gives positive feedback. | 8.1. Behavioural practice  4.1. Instruction on how to perform a behaviour  6.1. Demonstration of behaviour  4.4. Behavioural experiment  2.4. Self-monitoring outcomes of the behaviour | PowerPoint slides | Outcome expectations  Descriptive norm  Knowledge  Self-efficacy |
| **ACTION exercise review: Daily** **incidental activity goal**  **(8 min)** | Students:   - learn how to review goals which they have set last week; - learn how to identify barriers preventing them from being physically active and develop different strategies to overcome these barriers; - increase self-efficacy in overcoming barriers for PA. | - Facilitator projects questions regarding the daily incidental activity ACTION exercise from the previous week on the wall:   - What was the feeling after pursuing the goal? Did you achieve your goal?   - Were there some barriers on the way? If yes, how could you overcome the barriers next time?   - Ideas or tips? - Students interview each other on the questions. - After the pair discussions facilitator asks students | 1.2. Problem solving  2.2. Review behaviour goals/self-monitoring records  5.4. Monitoring of emotional consequences  2.2. Feedback on behaviour | PowerPoint slide  Workbook | Behavioural self-regulation  Self-efficacy  (Descriptive norms)  (Knowledge) |
| ***Exercise*** | ***Objectives*** | ***Activity description*** | ***BCTs for PA and/or SB*** | ***Materials*** | ***Determinants of PA and/or SB*** |
| **(cont.)** |  | to share key points, and then sums up the discussions.   - Facilitator makes sure that the discussion focuses on learning from the experiments and guides coping planning for goals that were not achieved. | 6.2. Social comparison  1.6. Discrepancy between current behaviour and goal |  |  |
| **PA map: Where to be physically active and how?**  **(15 min)** | Students:   - experiment a new way of being physically active by trying something new; - learn about different, low-price and easy PA opportunities, which require no special equipment; - notice that other students are also willing to be physically active or try something new; - get information about the LMI social media channels (Instagram and Facebook). | - Facilitator divides the students into four small groups, and each is appointed one setting / environment (home, school, city, digital). - On a large sheet of paper, students in each small group map by listing or drawing as many PA opportunities/sports/forms of physical activity as they can think of that could possibly be done in their respective setting. - The small groups present their list to the whole group. - After presentations, each student marks as many activity forms as s/he finds it would be possible to try in the different settings during the next week, and then picks the most inspiring and meaningful activity forms as the next week goal for themselves. - Facilitator introduces students with the intervention’s social media channels (Facebook and Instagram) and prompt students to follow the channel if they want. | 8.1. Behavioural practice  1.1. Goal setting  3.1. Social support (unspecified)  4.2. Information about antecedents | PowerPoint slide  Big empty paper sheets  Magnets  Marker pens | Descriptive norm  Knowledge  Self-efficacy  Social support  Environmental opportunities |
| **ACTION exercise: PA Experiment**  **& wrap-up of the session**  **(10 min + 5 min)** | Students:   - discover / are presented with low-priced and accessible PA opportunities , including know where to find LMI home workout videos; - set a PA experiment goal for the next week; - feel themselves optimistic about finding a new inspiring way of being physically | - Facilitator provides info about LMI workout videos (on [www.letsmoveit.fi](http://www.letsmoveit.fi), for the videos see <https://bit.ly/2J6btMm>) and where these videos can be found. - Facilitator provides students information on special offers from fitness studios / gyms / exercise clubs etc. which are located in the local neighbourhood of the school. - Students choose one new form of physical activity | 4.4.Behavioural experiments  8.1. Behavioural practice  1.1. Goal setting (behaviour)  1.4. Action planning |  | Behavioural self-regulation  Self efficacy  Knowledge  Social support |
| ***Exercise*** | ***Objectives*** | ***Activity description*** | ***BCTs for PA and/or SB*** | ***Materials*** | ***Determinants of PA and/or SB*** |
| **(cont.)** | active;   - form intention to try the new way of being physically active during next week. | (or one they have not tried in a long time) which they want to try/experiment during the following week.   - Students write their action plan down in the workbook or use a mobile app of their choice - Facilitator asks students also to assess how they feel after achieving the goal. - To help avoid unrealistic and unachievable goals, the facilitator points out that also a small experiment may be enough. - Facilitator guides the students to summarize for themselves the most important messages of the session by asking open ended questions:   - What is the level of my physical fitness?   - What kind of PA opportunities do I have? - What is my new PA experiment for the next week – my SMART plan? | 5.4. Monitoring of emotional consequences  2.3. Self-monitoring of behaviour  3.1. Social support (unspecified) *(prompted friends’ support in experiments)*  15.1. Verbal persuasion about capability |  | Environmental opportunities |
| “BE SMART” | SESSION 4  (45 min) | **Summary**   - **Critical assessment of unrealistic messages in media concerning PA and body images** - **Let’s Move It activity breaks** - **What is a SMART goal, and how to set a goal which follows principles of a SMART goal?** |  |  |  |
| **Opportunity for brief feedback**  **(2 min)** | Students:   - get a chance to give feedback and tell opinions about LMI program and facilitator behaviour; - have an opportunity to affect the way sessions will be held in the future. | - Facilitator asks for oral feedback on the program and on his/her working methods before the session starts. |  |  |  |
| **ACTION exercise review: PA experiment**  **(8 min)** | Students:   - develop their skills on self-evaluation of goal progress ; - learn the basics of coping planning; - share information about successful PA experiences; | - Facilitator places the PA maps completed at session 3 on the wall as a reminder. - In a short pair discussion and subsequent reporting back to the whole group, students review their success in PA experiments, identify barriers and ways to overcome these barriers | 1.5. Review behavioural goals  5.4. Monitoring of emotional consequences | PowerPoint slide  PA Map paper sheets | Behavioural self-regulation  Autonomous motivation (integrated |
| ***Exercise*** | ***Objectives*** | ***Activity description*** | ***BCTs for PA and/or SB*** | ***Materials*** | ***Determinants of PA and/or SB*** |
| **(cont.)** | - reflect the meaning of autonomous motivation in PA; - hear other students speak about their PA experiences (strengthens the change talk in the group and among the group participants). | within the whole group.   - Facilitator encourages students to speak about their reasons for choosing the specific PA experiment. - Facilitator asks students to share positive outcomes but also barriers and problems encountered with the experiments. - Facilitator helps students to find ways to overcome obstacles in the future. | 1.2. Problem solving  2.2. Feedback on behaviour  1.6. Discrepancy between current  behaviour and goal  10.4 Social reward | Magnets  Workbook | motivation regulation)  Outcome expectations  Self-efficacy |
| **Activity break**  **(5 min)** | Students:   - recall/know where to find LMI workout videos and how to use the videos at home; - reduce sitting time; - are motivated to ask other teachers to use activity break videos during school lessons. | - Facilitator shows where to find the LMI workout videos (www.letsmoveit.fi). - Facilitator presents the whole selection of videos and prompt students to ask their teachers to use videos in normal classroom settings. - Students collectively choose and then participate in LMI-activity break ([www.letsmoveit.fi](http://www.letsmoveit.fi/)). - Facilitator participates in break and encourage students in a positive way. | 4.1. Instruction on how to perform a behaviour  8.1. Behavioural practice  6.1. Demonstration of the behaviour  15.1. Verbal persuasion about capability | PowerPoint slide  Website  YouTube | Outcome expectations  Descriptive norm  Self-efficacy  Environmental opportunities |
| **Fitspiration & body image ideals: Critical assessment of popular myths**  **(15 min)** | Students:   - learn to assess critically unrealistic messages concerning PA and body images in the media (including e.g. heavy idealisation of extremely “fit” or muscular bodies); - form a broad range of positive outcome expectations of PA, including more than appearance; - understand the principles of safe training to prevent injuries; - understand that too much and too intensive PA can lead to various problems, and that PA should be increased with moderate, graded steps; | - Facilitator divides students into groups of five and gives each group a handout with a provocative image showing one prevalent PA misconception**,** either appearance or performance achievements. (“fitspiration”). - Based on the images students ponder on distorted views concerning typically body image in media and excess exercise, with guiding questions: - What is wrong in these pictures and texts when you are not a professional athlete? - How does an adolescent understand these kinds of messages? - What would be a safer and more responsible message? - How would you re-formulate the text that | 5.1. Information about health consequences  5.6. Information about emotional consequences  8.1. Information about antecedents  8.7. Graded task  12.5. Adding objects to the environment (videos) | PowerPoint slide  Handout with a provocative image and text | Self-efficacy  Autonomous motivation  Knowledge  Outcome expectations |
| ***Exercise*** | ***Objectives*** | ***Activity description*** | ***BCTs for PA and/or SB*** | ***Materials*** | ***Determinants of PA and/or SB*** |
| **(cont.)** | - recall the most important messages of LMI, especially Goal: well-being, not fatless body. | accompanies the photo, to be more responsible, safe, and humane?   - After the small group discussions, the facilitator highlights the importance of goal setting in a way that takes into account the student’s own starting level. During the debriefing, the facilitator tears the handout sheets into pieces, throwing them into bin, to memorably demonstrate that such extreme fitspiration quotes are virtually myths. For a detailed translation of this facilitator manual section, please see Table S6-c. - Facilitator emphasizes the multiple positive outcomes of PA, concentrating strongly on outcomes unrelated to appearance, and prompts students to set tasks that are initially easy, but that can be made increasingly difficult, yet achievable. |  |  |  |
| **ACTION exercise: Personal SMART goal and plan**  **+ wrap-up of the session**  **(15 min)** | Students:   - understand principles of setting good goals; - learn what is a SMART goal and understand its benefits; - learn how to set a SMART PA goal for themselves; - improve their goal setting and action planning skills. | - Facilitator presents two different PA goals, one of which follows SMART principles, the other not:   - SMART principles: specific, measurable, achievable, relevant and time-bound. - After a short small group discussion on which one is better and why, the students evaluate the presented goals in the whole group. - Facilitator leads the discussion and makes sure that students understand the meaning of SMART goal setting and the differences between effective and ineffective goal setting. - Instead of outcome goals (e.g., weight, muscle mass) facilitator underlines the usefulness and importance of setting (also) precise behavioural goals (e.g., increasing the number of exercise sessions) | 1.1. Goal setting (behaviour)  1.4. Action planning  2.3. Self-monitoring on behaviour (PA diary for the two last weeks)  5.4. Monitoring of emotional consequences (column in the PA diaries) | PowerPoint slides  Workbook (or mobile app)  Pencils | Behavioural self-regulation  Self-efficacy  Autonomous motivation |
| ***Exercise*** | ***Objectives*** | ***Activity description*** | ***BCTs for PA and/or SB*** | ***Materials*** | ***Determinants of PA and/or SB*** |
| **(cont.)** |  | - Facilitator highlights that a goal will more likely be achieved if it follows the SMART goal principles. - Working individually or in pairs, students set themselves a specific, measurable, achievable relevant and time-bound PA goal and write it down in their workbook (or in a mobile app). - Facilitator guides the group to summarize the most important topics of the week by asking open ended questions:   - What do you find from www.letsmoveit.fi?   - What kind of unrealistic illusions concerning body images and PA are presented in the media? - What kind of PA goal is good and effective? | 15.1. Verbal persuasion about capability |  |  |
| ”BARRIERS ON THE WAY?” | SESSION 5  (45 MIN) | **Summary:**   - **Meaning of social support when increasing activity in everyday life & tips to get it** - **How to identify and overcome barriers for being physically active** |  |  |  |
| **The most important messages from last week**  **(5 min)** | Students:   - recall the most important messages of the previous week. | - Avoiding pressuring feeling but showing genuine curiosity, the facilitator enquires what students remember from the previous week´s session. - Facilitator prompts for recollections of activity break (and online workout videos available on the LMI website), SMART goal setting, and illusions/misconceptions concerning body image and PA. |  | PowerPoint slides |  |
| **ACTION exercise review: Personal SMART goal & plan** | Students:   - strengthen their skills in goal achievement evaluation and understanding of SMART goal setting; - identify and learn to utilize PA facilitator; - learn ways to identify and overcome PA barriers and learn the basics of coping | - With the whole group, students review their SMART goal progress and achievement. - Students also identify any barriers and facilitating factors for PA goals. - Facilitator instructs students to discuss ways in which they could help overcome the barriers. - Students write down the barriers and solutions on the blackboard. | 1.5. Review behaviour goals  1.6. Discrepancy between current  behaviour and goal  1.2. Problem solving | PowerPoint slides  Blackboard | Behavioural self-regulation  Self-efficacy  Descriptive norm  Social support |
| ***Exercise*** | ***Objectives*** | ***Activity description*** | ***BCTs for PA and/or SB*** | ***Materials*** | ***Determinants of PA and/or SB*** |
| **(cont.)** | planning;   - learn new ways to get their circle of family, friends and acquaintances to support enactment of their PA plans; - consider putting the new ways into practice when needing social support | - Facilitator makes sure that one or multiple solutions for every barrier are generated, so that no barrier remains without any solution. - Facilitator highlights successful experiments in goal setting and action planning - Facilitator makes sure by prompting with open ended questions that at least 3 different forms of social support (from friends or family) have come up during the discussion (if not, facilitator asks e.g. questions such as “What kind of sports do your parents enjoy?”, with the intention of suggesting engaging in PA together). See Table S6-c. | 5.4. Monitoring of emotional consequences  3.2. Social support (practical)  3.3. Social support (emotional)  13.1. Identification self as a role model  6.3. Information about others’ approval  12.2 Restructuring the social environment  6.3 Information about others’ approval  10.4 Social reward |  | Autonomous motivation |
| **Problem solving: Coping Plan Consultants(20 minutes) (see also Hankonen et al., 2017^3^)** | Students:   - learn ways to identify and overcome their subjective PA barriers; - learn the concept of coping planning and understand the relevance of it; - learn how to re-build the social and physical environment in a way which supports achievement of PA goals; - get practical examples showing that typical barriers for PA among youth can almost always be overcome and various solutions can be found; - strengthen self-efficacy in overcoming | - Students form 4 small groups. - Facilitator gives each group a different case study of an adolescent who has barriers that prevent him/her from being physically active. - In the small groups, students identify the PA barriers and try to generate solutions. - Each small group presents their case and solutions to the whole group. - During the discussion, facilitator keeps the focus on problem solving instead of only identifying the barriers, and normalizes having various barriers in youth’s life. - The facilitator steers the conversation from general level to also the students’ personal level. | 1.2. Problem solving  4.2. Information about antecedents of behaviour  13.1 Identification of self as role model | PowerPoint slide  4 Case study sheets (problem solving)  Pencils | Self-efficacy  Behavioural self-regulation |
| ***Exercise*** | ***Objectives*** | ***Activity description*** | ***BCTs for PA and/or SB*** | ***Materials*** | ***Determinants of PA and/or SB*** |
| **(cont.)** | barriers: when “coaching” the imaginary person, students are able to both take the role of an “outside expert”, and focus on solutions rather than problems, thus enhancing their self-efficacy |  |  |  |  |
| **ACTION exercise:**  **PA action plan v. 2 + Coping planning**  **+ wrap-up of the session**  **(10 min)** | Students:   - learn ways to identify and overcome their personal PA barriers; - understand the relevance of coping planning when setting their next action plan; - set a personally meaningful PA action plan and make a coping plan; - be motivated to record their daily exercises in a PA diary. | - Students fill in the “Barriers and solutions of PA” exercise in their workbook (identify barriers, then match with suggested solutions) - Facilitator asks each student to set a personally meaningful SMART action plan in the workbook for the next two weeks. Facilitator also asks students to keep a record of their goal progress and eventual barriers. - Facilitator reminds that setting of SMART goals and intermittent self-monitoring increase the probability of success in goal attainment. It is emphasized that it is not expected that students will continue self-monitoring every week from now on, but rather use it as a tool, whenever they feel they want to check in how they are doing in terms of PA - Facilitator guides the group to summarize the most important topics of the week by asking open ended questions:   - How can you make sure that you achieve your goal?   - How can you overcome the barriers on your way? | 1.1. Goal setting (behaviour)  1.4. Action planning   - 1. Problem solving   2.3. Self-monitoring | PowerPoint slide  Workbook  Pencils | Behavioural self-regulation  Self-efficacy |
| “THE JOURNEY CONTINUES” | SESSION 6  (45 min) | **Summary:**   - **Revisiting students’ PA identity and their thoughts about (excessive) sitting at the moment** - **Repeat the main messages of LMI** - **PA action plan for the next 4-week period** |  |  |  |
| ***Exercise*** | ***Objectives*** | ***Activity description*** | ***BCTs for PA and/or SB*** | ***Materials*** | ***Determinants of PA and/or SB*** |
| **ACTION exercise review:**  **PA action plan v.2**  **+ Coping planning**  **+ Filling up a sitting reduction checklist**  **(10 min)** | Students:   - reflect/assess their goal achievements and understand better their barriers for PA; - gather ideas for novel means to overcome the barriers; - learn to think how to reduce their sitting outside of school setting. | - Students discuss the previous week’s action exercise with the whole group. - Facilitator guides the conversation with following questions:   - What kind of issues make it easier for you to follow /achieve the action plan?   - What kind of issues make it more difficult?   - What kind of solutions did you come up with? - Facilitator focuses on action plans that have been successfully achieved and on helping students to engage with coping planning and to find solutions to overcome different barriers. - Facilitator draws attention already to the future and explains that the next action plan will be planned for the next 4 weeks. - After reviewing/assessing the action plan students fill up “Sitting reduction checklist” in their workbooks. They choose and mark one or more easy and personally meaningful ways to reduce their sitting in daily life (at home, in school, at work or in public transportation) also after this last LMI session. - Facilitator encourages students to share their experiences of prolonged sitting (e.g. “Have you noticed some negative consequences of prolonged sitting? You have? Would you like to tell more about them? How about the rest of you?”). | 1.5. Review of behavioural goals  1.6. Discrepancy between current  behaviour and goal  1.2. Problem solving  1.4. Action planning (only SB)  15.3. Focus on past success  6.3 Information about others’ approval  16.3 Vicarious consequences | PowerPoint slides  Workbook  Pencils | Behavioural self-regulation (also SB)  Autonomous motivation (also SB) |
| **Main messages of Let’s Move It program (opinion line exercise)**  **(10 min)** | Students:   - review the main messages of LMI (“the 6 LMI theses”) - identify implicit, potentially harmful assumptions, make them explicit and change them into more fit-for-purpose assumptions that will promote PA motivation; - recall the multiple positive | - Facilitator projects the six main theses of the program on the wall one at a time, and asks the students to place themselves on an imaginary opinion line based on how much they agree or disagree with the message. - After each thesis, facilitator asks 1-2 students give reasons for their position on the opinion line and tries to steer the conversation to a positive direction in the light of PA perceptions. | 4.2. Information about antecedents of behaviour  5.1. Information about health consequences | PowerPoint slides  PA benefit cards | Self-efficacy  Knowledge  Outcome expectations  Autonomous motivation |
| ***Exercise*** | ***Objectives*** | ***Activity description*** | ***BCTs for PA and/or SB*** | ***Materials*** | ***Determinants of PA and/or SB*** |
| **(cont.)** | consequences of PA and become aware of their own personal reasons; broaden perceptions on PA;   - have an opportunity to interrupt their sitting. | - When facilitator projects the ‘Know what moves you’ message on the wall, students are asked to pick the most important and meaningful reasons for themselves to be physically active. - Facilitator highlights that only students themselves can know, what the important reasons are for themselves. | 5.6. Information about emotional consequences  5.3. Information about social and environmental consequences  5.2. Salience of consequences  6.3 Information about others’ approval  16.3 Vicarious consequences  15.3. Focus on past success  15.1. Verbal persuasion about capability |  | Descriptive norm |
| **PA identity continuum exercise**  **(10 min)** | Students:   - work to change their PA identity; - Continue broadening their perceptions on what PA entails; - examine their PA identity and realize that it is not a matter of either athlete or sedentary but a more multi-faceted phenomenon, and that everyone already has ways of being physically active (“We are all entitled to activity”); - produce pro-PA change talk; - understand that the LMI program is not aiming to make everyone a fitness freak but that even small changes count (“Adding any movement is good!”) | - The facilitator presents both the dichotomous and the more fine-grained model of PA identities (shown already on Session 1, PA identity continuum exercise, see Table 6-b). - Students are presented with questions on how they experience PA, and how they see their relationship with PA / different forms of PA will continue after the program has ended. - Discussion is conducted first in pairs and then with the whole group. - The facilitator aims to steer the conversation towards positive changes and to where students want to move on the line with their PA in the future (both types and amount of PA). | 13.5. Identity associated with changed behaviour  15.1. Verbal persuasion about capability  13.2 Framing/Reframing  15.3. Focus on past success | PowerPoint slide | Knowledge  Outcome expectations  Self-efficacy  Autonomous motivation (integrated motivation regulation) |
| ***Exercise*** | ***Objectives*** | ***Activity description*** | ***BCTs for PA and/or SB*** | ***Materials*** | ***Determinants of PA and/or SB*** |
| **(cont.)** |  | - The facilitator emphasizes the benefits of even small changes and pre-existing forms of PA, and that each of us already is active, i.e. is an “insider” in at least some forms. - The facilitator steers discussion to promote understanding of daily chores and incidental activity as PA. - The facilitator makes sure that students understand that it’s normal to have different amounts and types of PA in different phases of life and that even after a break, one can still resume being active, i.e., “relapses” or pauses in regular PA should not be interpreted as signs of being inactive for good. | 5.1. Information about health consequences  15.3. Focus on past success  4.3. Re-attribution |  |  |
| **Behaviour change skills in action**  **(5 min)** | Students:   - get an idea how they can self-manage and regulate other areas of their behaviour with the help of the behaviour change skills they have learnt in the LMI (e.g. behavioural self-regulation cycle); - understand how the LMI program is built and how the variety of exercises form a coherent whole. | - Facilitator projects a picture about behaviour change techniques which have been used on the wall (picture from workbook, see Supplementary Figure S3) and makes visible the link between these techniques and the LMI exercises. - Facilitator steers the students’ thoughts to the future and to how they could use the techniques in other areas of their life (e.g. applying for a job). - Facilitator points out that motivation is nothing mysterious but there are certain factors that support the development of motivation. Facilitator highlights that people can enhance their own motivation in many ways (e.g. by thinking/imagining/anticipating personally meaningful consequences of PA). | 4.2. Information about antecedents of behaviour  4.1. Instruction on how to perform a behaviour  15.3. Focus on past success | PowerPoint slide  Workbook | Self-efficacy  Autonomous motivation |
| **ACTION exercise: a SMART action plan for 4 weeks**  **(5 min)** | Students:   - set an interesting PA goal and action plan for the next 4 weeks with the help of coping planning sheet from last weeks; | - Facilitator instructs setting an inspiring PA goal and creating an action plan, following the principles and practices from session 4 exercise “Own SMART goal and plan” for the next 4 weeks, with the help of coping planning sheet from last weeks (students can choose either paper sheet or | 1.4. Action planning  1.1. Goal setting (behaviour)  8.7. Graded tasks | PowerPoint slide  SMART-action plan sheets | Behavioural self-regulation  Autonomous motivation |
| ***Exercise*** | ***Objectives*** | ***Activity description*** | ***BCTs for PA and/or SB*** | ***Materials*** | ***Determinants of PA and/or SB*** |
| **(cont.)** | - strengthen skills for long-term goal setting. | e.g. mobile app)   - Facilitator highlights the importance of goal setting in a way which takes students’ own current PA level into consideration. - Facilitator points out that students do action planning for themselves, not for the facilitator or school. | 2.3. Self-monitoring of behaviour | Pencils | Self-efficacy |
| **Afterword**  **(5 min)** | Students:   - get positive feedback as a whole group and as individual group member who are important part of the group; - gain awareness about LMI social media channels (Instagram and Facebook) to be able to follow posts concerning PA to e.g. get more information and tips, and to keep up their motivation. | - Facilitator thanks the group by reviewing group accomplishments, reflecting on student engagement and acknowledging the importance of each student and their mutual support to each other. - Facilitator reminds the group of LMI social media channels and writes the addresses also on the blackboard. - Facilitator gives students information on a booster session which will take place after a few months. In the booster session students will have an opportunity to reflect on their relationship with PA again. | 10.4 Social reward  4.1 Instruction on how to perform the behaviour | PowerPoint slide | Positive group climate  Self-efficacy |
|  | BOOSTER SESSION  (45 min) | **Summary:**   - **Students revisit/examine their PA identity post-LMI program** - **Reviewing current personally important and meaningful reasons for PA** - **Reviewing sedentary behaviour reduction benefits and tips** - **Recognize that they are already being physically active in many ways** |  |  |  |
| **PA identity continuum (recap) (5 min)** | Students:   - recollect that PA is not just PE at school or vigorous exercise / sweating, and that various types of PA exist; - remember, that all people are made to | - Facilitator again uses the two slides showing a dichotomous view of PA identity and then the more fine-grained, flexible model that distinguishes “guests”, “tourists”, “regular visitors” and “insiders” (see Table S6-b). | 13.5. Identity associated with changed behaviour | PowerPoint slides | Knowledge  Outcome expectations  Self-efficacy |
| ***Exercise*** | ***Objectives*** | ***Activity description*** | ***BCTs for PA and/or SB*** | ***Materials*** | ***Determinants of PA and/or SB*** |
| **(cont.)** | be physically active;   - understand that the purpose of the LMI program was not to turn them into “fitness freaks” – that even small improvements on the continuum count. | - Facilitator leads discussion towards common understanding that there are many ways to be active and you can choose the ones you like - Facilitator again highlights that there is a sporty person inside every one of us, that doing PA is natural for humans, that even small improvements benefit - Facilitator helps identify existing forms of PA in students’ current daily lives | 15.1. Verbal persuasion about capability  5.1. Information about health consequences  15.3. Focus on past success |  | Autonomous motivation (integrated motivation regulation) |
| **Reviewing personally important reasons for PA (recap)**  **(8 min)** | Students:   - re-visit their thoughts on the most important personally meaningful reasons for them to be physically active; reflect whether there have been any changes - learn about/recap various positive consequences of PA; - hear other students to talk about PA and its consequences in a positive way; - remember that light/moderate activity and daily incidental activity also benefit - it doesn’t have to be a matter of everything or nothing | - Facilitator projects a slide on positive consequences of PA on the wall (partially based on the benefits shown during the Identifying Personal Motives Group Activity) - Students get to say out loud the reasons important for them so that others can hear them too, and write them down on a separate PA diary template. - Facilitator prompts students to consider whether there have been any changes after the LMI program. | 5.1. Information about health consequences  5.2. Salience of consequences  5.3. Information about social and environmental consequences  5.4. Information about emotional consequences  6.3 Information about others’ approval  16.3 Vicarious consequences | PowerPoint slides | Descriptive norms  Autonomous motivation  Outcome expectations |
| **Sitting reduction checklist (5 min)** | Students:   - remind themselves of the consequences of excessive sitting, both personally and in general; - increase their motivation for reducing sedentary behaviour; - recall that incidental activity can reduce | - A poster on consequences of sitting is shown on the screen. - Facilitator guides a discussion on consequences of excessive sitting and about sedentary behaviour reduction. - Students vote for their favourite way of SB reduction from a checklist presented on the screen. | 5.1. Information about health  consequences  4.1. Instruction on how to perform the behaviour | PowerPoint slides | Outcome expectations  Autonomous motivation  Descriptive norm |
| ***Exercise*** | ***Objectives*** | ***Activity description*** | ***BCTs for PA and/or SB*** | ***Materials*** | ***Determinants of PA and/or SB*** |
|  | the harms related to excessive sitting;   - brainstorm on situations in which sedentary behaviour reduction could become a habit. |  | 8.2 Behaviour substitution  16.3 Vicarious consequences |  |  |
| **Problem solving: Coping Plan Consultants, whole group (10 min)** | Students:   - strengthen their skills in identifying and overcoming their subjective PA barriers; - improve their skills in coping planning and understand the relevance of it; - increase self-efficacy in their ability to overcome barriers; - hear others talk about barriers and solutions. | - A case description is presented of a young person facing challenges for regular PA. - Students get a role as the PA counsellors whose task is to identify the challenges and barriers and find solutions together as a group. | 1.2. Problem solving  4.2. Information about antecedents of behaviour  13.1. Identification self as a role model | PowerPoint slides | Self-efficacy  Behavioural self-regulation |
| **ACTION exercise: PA map and action plan (12 min)**  **+ wrap-up of the session (1 min)** | Students:   - get reminded of low cost and accessible PA and exercise facilities and opportunities in their neighbourhood; - strengthen their goal setting and planning skills for long-term goals; - remember that barriers can be tackled proactively. | - Students fill in their longer-term PA goals and action plans in the workbook. - The facilitator presents simple exemplary goals and action plans, and reminds that goals should be adjusted to one’s own level of activity, and be SMART. - Students fill out coping plans to deal with potential anticipated barriers. | 1.4. Action planning  2.3. Self-monitoring of behaviour  1.2. Problem solving (coping planning and relapse prevention included) | PowerPoint slides  Old PA maps  A separate PA diary and Overcoming barriers activity printed for everyone | Self-efficacy  Behavioural self-regulation |
| **Wrap-up of the session & goodbye** | Students   - get a positive last impression | - The facilitator thanks the students for the session, focusing on how well they have been engaged, and reminds again of the [www.letsmoveit.fi](http://www.letsmoveit.fi) resources - Facilitator suggests that the students can continue doing their PA plans together with their friends if they want - Facilitator invites eventual questions, and thanks once more for participation. | 10.4 Social reward |  | Positive group climate  Self-efficacy |

^1^  Unruh, D. R. (1980). The Nature of Social Worlds. *Pacific Sociological Review*, 23(3), 271–296. https://doi.org/10.2307/1388823

^2^ Note. This Girl Can video was not played in all batches due to feedback from teachers.

^3^ Hankonen, N., Heino, M. T. J., Hynynen, S.-T., Laine, H., Araújo-Soares, V., Sniehotta, F. F., … Haukkala, A. (2017). Randomised controlled feasibility study of a school-based multi-level intervention to increase physical activity and decrease sedentary behaviour among vocational school students. *International Journal of Behavioural Nutrition and Physical Activity*, 14(1), 37. https://doi.org/10.1186/s12966-017-0484-0

Note. For activities such as planning, the table refers by default to PA, unless SB is explicitly stated.

**Table S6-b. PA identity continuum exercise, figure shown during the exercise (Session 1, Session 6, booster).**

| The following images were shown to the students, demonstrating (a) a dichotomous, stable view of people as active or inactive, and (b) a more fine-grained view with different trajectories, enabling changes from different groups to another, and reminding that all of us are “insiders” in some, and “guests” in other forms of PA.  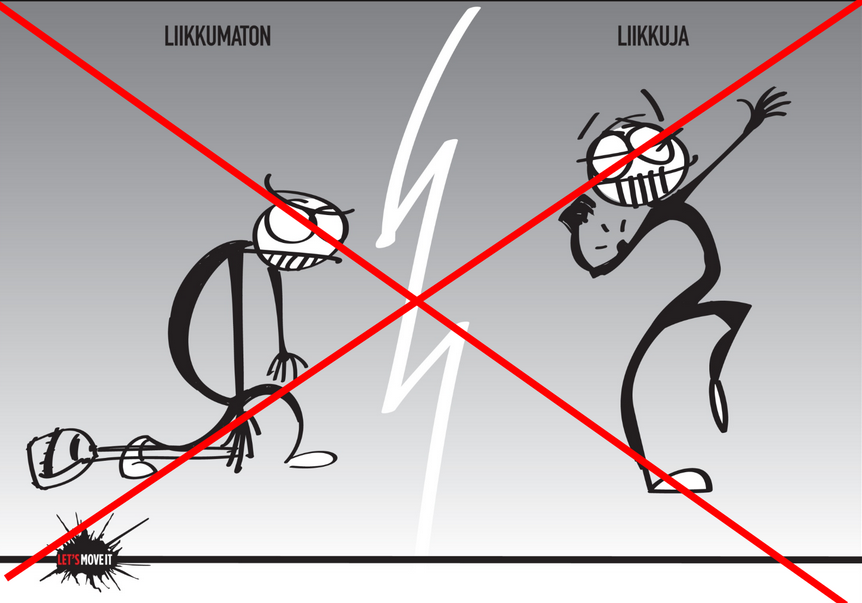 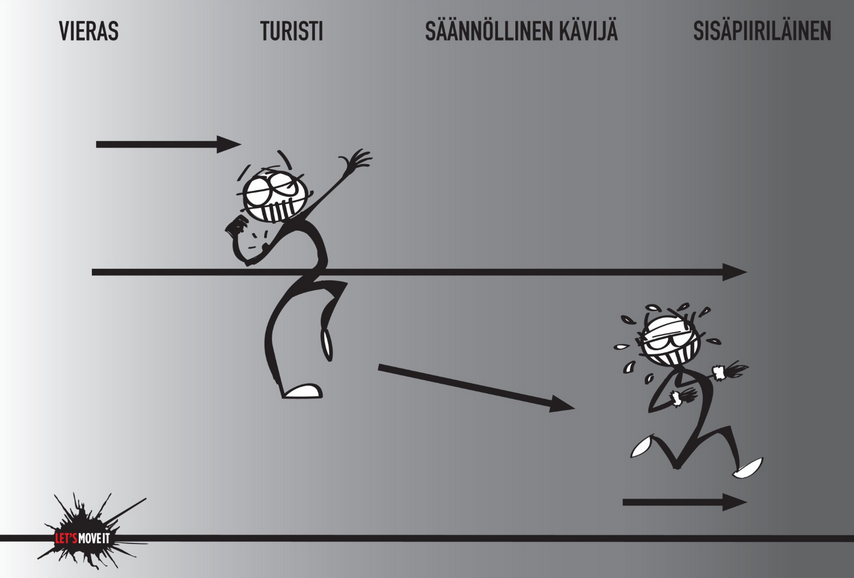 |
| --- |

**Table S6-c. Detailed translations of facilitator manual for key messages in selected exercises.**

| **Fitspiration & body image ideals: Critical assessment of popular myths (on Session 4)**   - Points beneficial to be raised during group discussion in regards to physical activity Myth #1: “Tiredness is weakness ” - The discussed poster shows a muscular male bodybuilder lifting weights with angry facial expressions and the text reads: “Are you panting? Are you sweaty? Are you exhausted? Do you feel sick? Do you feel tired? When your body is about to give up, get angry about getting tired. Tiredness is weakness. Anger makes you work out harder!” - The image says that only extreme exercise can be considered exercise, but this is not true. - It is good to have plans and goals but listening to one’s body is important. - What is the problem with weight-loss reality TV shows? People with little exercise experience and low fitness levels go straight to heavy, vigorous training. What do you think about that? - It is good to add quantity first and intensity second. Recovery training is also important! - People with very little workout experience go all out right from the start – this message is too prevalent. - If you ignore tiredness when exercising, what can it lead to? 1) Decreased alertness and increased accident risk 2) Injuries 3) Depressive symptoms 4) What else? - Suggestions on how to alter the text: “If tired, rest”, “Tiredness is normal, do not force yourself to exercise” - Points beneficial to be raised in regards to physical activity Myth #2: “Muscular is the new skinny” - The poster shows a female fitness athlete with a six-pack posing and says: “Hard work is the key to success. Muscular is the new skinny.” - The person in the image is a fitness professional and even she has to train safe. They fast for the photo shoots and the image might have been edited. This may be OK for a professional athlete but is a six-pack achievable for a non-professional? - It is common to suggest that one’s figure and looks are the only reason to exercise. But in fact, muscularity or thinness (“body modification”) is not the most important goal as PA has many other pros that are unfortunately often forgotten. - Muscularity or thinness do not guarantee healthiness. - Research has even shown that a physically active overweight person is healthier and lives longer than an inactive skinny person. - Who recognizes the idea of doing PA for looks? Who defines what looks good? To what extent do you think a person can do PA for looks? What other PA-related pros can you come up with? - Is it natural to have a completely fatless body? (especially for women in terms of hormonal balance) - Suggestions on how to alter the text: “Hard work is the key to success but you can define what success means to you (not a six-pack but e.g., skills)”. |
| --- |
| **ACTION exercise review: SMART goal (on Session 5) – section about how to obtain social support for PA**   - The facilitator makes sure that at least three different ideas for obtaining different forms of social support are brought up in the discussion. These can be expressed by the students spontaneously, or elicited through questions posed by the facilitator. - Examples: - More active way of spending time together, i.e. instead of sitting at a café or on a computer, you can go shopping/play footbag, etc. - Suggesting a new form of PA to a friend, e.g. you can combine chatting with gym training. - For encouragement, facilitator can mention that based on research it is known that a majority of the youth would like to be more physically active, so a friend would be pleased about the suggestion, too. - Set a PA date, e.g. an evening walk, biking to school, etc., in order not to divert from the plan. - Ask what kind of sports students’ parents would like to do – many parents want to do something with their children. Suggest doing evening yoga to your mom or doing LMI strength exercises with your dad. - Set an example for your younger siblings: ask them to join you to do the LMI home workout videos! |
