# Supplementary material for: Changing activity behaviours in vocational school students: the stepwise development and optimised content of the ‘let’s move it’ intervention
Source: Health Psychol Behav Med. 2020 Sep 27;8(1):440–60. doi: 10.1080/21642850.2020.1813036 (PMC8114352; doi:10.1080/21642850.2020.1813036)
Supplement: Supplemental Material [file RHPB_A_1813036_SM8281.zip › suppl_data/S_Table_S7_Determinants_BCTs_Poster_and_standing_tri_fold_brochure_description_tables-.docx]

**Table S7. Determinants & BCTs: Poster and standing tri-fold brochure description tables**

|  | **Objectives** | **Key BCTs (based on BCTT v1)** | **Determinants** |
| --- | --- | --- | --- |
| **POSTERS** | | | |
| **Awareness (9 posters)** | Students   - understand that also a little PA is beneficial - become aware, due to the attractive and funny way of presentation (i.e. “I thought my life was perfect before I got up, Best wishes, Homo Erectus”) of PA and the Let’s Move It principles - increase positive attitudes of engaging in PA | 5.2. Salience of consequences  6.3 Information about others’ approval | - Norms - Autonomous motivation - Self-efficacy |
| **Info on benefits (6 posters)** | Students   - identify different benefits of physical activity (i.e. “Exercise gives you a lot of energy”) - become aware of the varied, also short-term benefits of PA | 5.1. Information about health consequences  5.2. Salience of consequences  5.6. Information about emotional consequences  5.3. Information about social and environmental consequences | - Knowledge - Outcome expectations |
| **Tips on how to make one’s day more active / reduce sitting time (5 posters)** | Students   - get easy tips on how to make their days a bit more active or how they can reduce passive time. - understand that also small movements count | 4.1. Instruction on how to perform the behaviour  6.1. Demonstration of the behaviour  8.1. Behavioural practice and rehearsal | - Self-efficacy |
| **STANDING TRI-FOLD BROCHURES (set on cafeteria tables)** | | | |
| **1. How to motivate oneself and benefits of PA** | Students   - get tips on how they could motivate themselves for PA | 3.1 Social support  5.1. Information about health consequences | - Autonomous motivation - Outcome expectations - Knowledge |
| **2. Finding reasons to be active** | Students   - think about personally important reasons to be (more) active | 5.1. Information about health consequences  5.6. Information about emotional consequences | - Autonomous motivation - Outcome expectations - Knowledge |
| **3. Tackling barriers** | Students   - identify or analyse the possible barriers for PA and identify different strategies to overcome these barriers. | 1.2. Problem solving  5.1. Information about health consequences | - Self-efficacy - Self-regulation (problem solving) - Autonomous motivation |
| **4. Goal setting and monitoring** | Students   - set an achievable PA goal and keep on track how active they really are with the help of PA diary or mobile application. | 1.1. Goal setting (behaviour)  1.5. Review behavioural goals  2.3. Self-monitoring of behaviour  5.4. Monitoring of emotional consequences  8.7. Graded tasks | - Self-regulation (self-monitoring, goal setting) - Knowledge (What are the benefits of self-monitoring?) |
|  | **Objectives** | **Key BCTs (based on BCTT v1)** | **Determinants** |
| **5. Ways to reduce SB** | Students   - think about benefits of reduced passive time - set an easy MPA goal integrated in daily activities | 5.1 Information about health consequences  5.6. Information on emotional consequences  1.1. Goal setting (behaviour) | - Knowledge - Self-regulation (self-monitoring, goal setting) |
| **6. SMART goal setting** | Students   - set a SMART PA goal | 1.1. Goal setting (behaviour)  8.7. Graded tasks | - Self-regulation (self-monitoring, goal setting) - Self-efficacy |
| **7. Safe PA; goal setting** | Students   - pay attention also to injury prevention (i.e. they do not exercise when sick or tired) - make their PA goals more reasonable | 8.7. Graded tasks  4.1. Instruction on how to perform the behaviour | - Knowledge (injury prevention) - Self-efficacy - Autonomous motivation |
| **8. Finding reasons to be active** | Students   - think about important reasons for themselves to be physically active - get tips how to maintain enhanced PA level | 5.1. Information about health consequences  5.6. Information about emotional consequences | - Autonomous motivation - Outcome expectations |
| **9. Info on incidental activity** | Students   - understand the importance and usefulness daily PA (integrated in normal activities) - see inspiring and fun ways of being active during weekdays | 4.1. Instruction on how to perform the behaviour  5.1. Information about health consequences | - Self-efficacy - Knowledge |
| **10. Tackling barriers; Info on mobile apps** | Students   - get tips on what kind of mobile phone apps they can use to enhance their physical activity - understand how to overcome possible barriers of PA | 4.1. Instruction on how to perform the behaviour | - Knowledge - Self-regulation |
| **11. Info on incidental activity)** | Students   - set an easy MPA goal as part of one’s daily life - understand how to overcome possible barriers of PA (especially lack of time) | 1.1. Goal setting (behaviour)  4.1. Instruction on how to perform the behaviour | - Knowledge - Self-regulation (coping planning, goal setting) |
| **12. Info on benefits of PA** | Students   - remember the positive health consequences of PA | 5.1. Information about health consequences | - Knowledge - Outcome expectations |
