# Supplementary material for: Changing activity behaviours in vocational school students: the stepwise development and optimised content of the ‘let’s move it’ intervention
Source: Health Psychol Behav Med. 2020 Sep 27;8(1):440–60. doi: 10.1080/21642850.2020.1813036 (PMC8114352; doi:10.1080/21642850.2020.1813036)
Supplement: Supplemental Material [file RHPB_A_1813036_SM8281.zip › suppl_data/S_Table_S8_Feedback_or_challenges_identified_optimisation-.docx]

**Supplementary table S8.**  Feedback or challenges identified in the feasibility study and resulting intervention optimisation

| **Area of challenge** | **Source(s)** | **Challenge identified / feedback** | **Suggested optimisation** | **Enacted** |
| --- | --- | --- | --- | --- |
| Program  structure | Student  interviews  Researcher field notes | Intervention sessions too rushed / too short for more in-depth discussions on important topics | Extend the sessions 45 -> 60 -> 75 min | Session 1 to 60 min; for other sessions extension not feasible due to school curriculum constraints |
| Program structure | Student interviews | Students felt that the interval between sessions was too short | Frequency of meetings max 1 per week -> program to extend for six weeks | Yes |
| Program structure | Field notes  Attendance records | Students often miss classes, therefore do not receive the whole intervention | Workbook with all materials provided  Easily accessible materials on the internet  Additional solutions needed to make sure that the students actually attend to these materials | Yes  Yes  Not found |
| Curricular context | Student interviews  Fieldnotes | In vocational school, students never have homework -> how to get them do it? | Homework competition?  Public commitment?  Any other strategies? | No feasible solution identified |
| Session  structure | Student questionnaire  Field notes | Students lose their concentration quickly, students want breaks | If sessions extended -> need for actual breaks in teaching every 45 min  Purposeful activity variability / breaks within each single session | Yes |
| **Area of challenge** | **Source(s)** | **Challenge identified / feedback** | **Suggested optimisation** | **Enacted** |
| Communication strategy | HE-teacher discussions  Field notes | Often in vocational schools, students are used to being told what to do instead of being offered choices and opportunities to do something for themselves. It would require more repetition for a longer duration for them to adapt to a new teaching method where they are treated as agents of their own learning. | Train all teachers involved with the intervention group students the basics of an autonomy supportive communication style | Not feasible |
| Communication strategy | HE-teacher discussions  Field notes | Instructions on intervention activities need to be more precise & received and understood by the students | Make the instructions easier to follow and create a habit of students quietly listening while standing up.  Give the instructions first and then provide rationale. | Yes (some of the pair discussions or tasks were done standing up)  Yes. |
| Communication strategy | HE-teacher discussions  Field notes | Too much freedom was offered to the students when they were asked to do the intervention activities outside of LMI program sessions | Make intervention activities outside the sessions a prerequisite part of the HE-course  Communicate more clearly (yet in an autonomy supportive way) that extra-curricular activities are an integral part of the program | Not feasible  Yes |
| Communication strategy / Session content | Student interviews | Even after the program, students report many extrinsic motivators (e.g., ideal body image concerns, need to look good) | Add intervention content to support other types, non-extrinsic motivational goals, and add content criticizing body image pressures.  Explain briefly and in lay terms how quality of motivation relates to physical activity behaviour.  Pay selective attention to change talk that implies autonomous motivation. | Yes  No (not feasible in the time given)  No |
| **Area of challenge** | **Source(s)** | **Challenge identified / feedback** | **Suggested optimisation** | **Enacted** |
| Session content | Student interviews  Field notes | Too tight timetable and feeling hurried (this is a recurring theme in the interviews) | Prolong introductory session 1 to 60-90 minutes and include activities from session 2.  Move content from session 6 to session 2.  Prolong the whole program by 15-45 minutes. | Yes, yes 60 min  Yes  Yes, 15 min |
| Session content | Discussion with  HE-teacher | More detailed information on the health consequences of PA and SB needed | A 10-minute mini lecture on the consequences of physical activity to be delivered on the second intervention session | Yes |
| Session content | Field notes | Students are unable to estimate their sedentary time which makes it difficult to motivate them to decrease their sedentary time | Offer students feedback on the Hookie accelerometry data that was measured at baseline | Yes |
| Session content | Student interviews | Students often mention back and neck pain and that they often overlook or forget stretching and active recovery | Stretching and active recovery to be discussed in the sessions | Yes, partly |
| Session content | Student interviews | Some students felt they did not learn much new or get many insights during the LMI lessons. They were interested in a mini lecture given on principles of how physical condition develops | Include targeted information pitches with interesting content (sitting reduction, development of physical condition…) | Yes, partly (not feasible within time resources to add many) |
| Session content | Student interviews | Some students felt that there was too much sitting during the LMI lessons | Increase the number of (physically) activating participatory exercises  Change paper and pencil exercises to oral / group exercises | Yes  Yes |
| **Area of challenge** | **Source(s)** | **Challenge identified / feedback** | **Suggested optimisation** | **Enacted** |
| Session content | Field notes | Not all students did the behavioural experimenting with novel physical activities | Skip behavioural experimenting  Make experimenting obligatory – choose from three options  Replace with a simple goal setting exercise (checklist)  Provide better rationale for experimenting / lower the bar for experiments | No  No  Yes  Yes |
| Session content | Student interviews | Action planning was perceived to be difficult | Include more time for working on own action plans | Yes, a little |
| Session content | Facilitator observation | Bullseye exercise for analysing current lifestyle produces sustain talk | Remove the bullseye exercise from group discussion  Provide an optional written exercise in workbook | Yes  Yes |
| Session contents | Student interviews | Students describe seasonal variation in amount of PA: becoming active in summer and forgetting PA in the winter / autumn | Seasonal physical activity in meetings or workbook?  If seasonal decreases in PA come up in student discussions, it was addressed | No  Yes |
| Session content | Student interviews | Students did not always understand the purpose of the coping planning group exercise or could not link it with their own situation | During the exercise, facilitate a discussion on actual situations the students have experienced that have called for coping planning | Yes |
| Session content / Order of content | Student interviews | Fitness test on the sixth meeting was an eye opener to the students with regards to their own physical condition | As a wake-up call, a not-too-serious fitness test should be included in the start of the program before students begin with goal setting and planning | Yes, to the 3^rd^ session |
| **Area of challenge** | **Source(s)** | **Challenge identified / feedback** | **Suggested optimisation** | **Enacted** |
| Session content / Order of content | Student interviews | Goal setting was perceived to be difficult and boring | Practice goal setting e.g., in the 3rd session by using the healthy habit checklist -> SMART goal setting and planning only after the checklist exercise has been done.  Distribute goal setting exercises across the intervention, starting small from early sessions, proceeding to more detailed goal setting/action planning toward the end  Create a more interactive goal setting by using goal cards and group discussion. | Yes, partly  Yes  Not feasible |
| Session content / Materials | Student interviews,  Field notes | Coping planning exercise “Volitional help sheet” (Armitage et al) did not work with this target group. Options maybe were not relevant? | Make the Volitional help sheet optional in the workbook  With graphic design, modify the Volitional help sheet to make it more understandable and visually nicer looking  Produce a more relevant coping planning exercise by including barrier examples from student interviews and improve wordings | No  Yes  Yes |
| Materials | Student interviews | Students expect more concrete ideas and suggestions of goals. If left to set goals independently, they either will not set any or will set goals that they perceive “too big” for themselves | In the student workbook, provide a checklist on healthy habits as a simplified goal setting activity. Healthy habits to include PA, SR, diet, rest.  Provide a slide with example goals, with different PA examples depending on season (winter / summer sports)  SMART-slide demonstrating examples of SMART goal setting by two example imaginary persons | Yes, for SR  Yes  Yes  Yes |
| **Area of challenge** | **Source(s)** | **Challenge identified / feedback** | **Suggested optimisation** | **Enacted** |
| Materials | Student interviews | Students expect more concrete ideas and suggestions for PA. | In the student workbook, provide examples of gym-based and home-based training programs. Not everyone will be inspired by workout videos. | Yes, basic gym workout program |
| Materials | Student interviews  Steering group | There might be a risk for some students of excessive training or restricted eating which could lead to eating disorders | In the workbook, include information on the risks of excessive training and eating disorder  Add overall attention to injury prevention | Yes  Yes |
| Materials | Student interviews |  | Include case stories that students can identify with of young people who have changed the way they think about and experience physical activity |  |
| Boosters | Facilitator observation | Students did not answer telephone / mobile phone calls, hence personal calls are not feasible in delivering boosters | Booster needs to be delivered at a face-to-face group meeting where the whole group could be reached at the same time | Yes |
| The program website | Student interviews  LifeGuide program records | Students did not visit the program website. The reasons mentioned in the interviews: difficult access, uninteresting material, outdated / unappealing design | Workbook (hard copy) with all materials provided | Yes |
