# Supplementary figures and images for: Changing activity behaviours in vocational school students: the stepwise development and optimised content of the ‘let’s move it’ intervention
Source: Health Psychol Behav Med. 2020 Sep 27;8(1):440–60. doi: 10.1080/21642850.2020.1813036 (PMC8114352; doi:10.1080/21642850.2020.1813036)

**Supplementary figure S1.** Map of social influences on students’ physical activity.

**
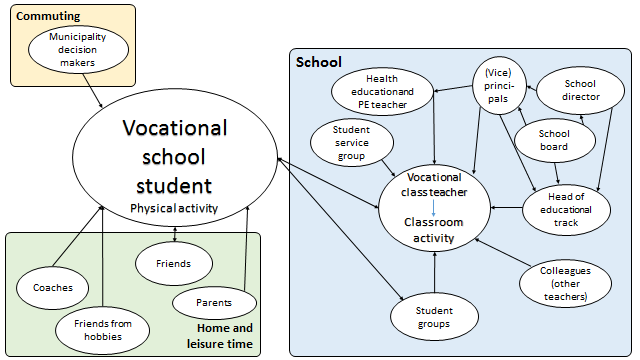
**

Supplement: Supplemental Material [file RHPB_A_1813036_SM8281.zip › suppl_data/S_Figure_S1_Map_of_social_influences_on_students_physical_activity-.docx]
